# Interactions between Melanin Enzymes and Their Atypical Recruitment to the Secretory Pathway by Palmitoylation

**DOI:** 10.1128/mBio.01925-16

**Published:** 2016-11-22

**Authors:** Srijana Upadhyay, Xinping Xu, Xiaorong Lin

**Affiliations:** Department of Biology, Texas A&M University, College Station, Texas, USA

## Abstract

Melanins are biopolymers that confer coloration and protection to the host organism against biotic or abiotic insults. The level of protection offered by melanin depends on its biosynthesis and its subcellular localization. Previously, we discovered that *Aspergillus fumigatus* compartmentalizes melanization in endosomes by recruiting all melanin enzymes to the secretory pathway. Surprisingly, although two laccases involved in the late steps of melanization are conventional secretory proteins, the four enzymes involved in the early steps of melanization lack a signal peptide or a transmembrane domain and are thus considered “atypical” secretory proteins. In this work, we found interactions among melanin enzymes and all melanin enzymes formed protein complexes. Surprisingly, the formation of protein complexes by melanin enzymes was not critical for their trafficking to the endosomal system. By palmitoylation profiling and biochemical analyses, we discovered that all four early melanin enzymes were strongly palmitoylated during conidiation. However, only the polyketide synthase (PKS) Alb1 was strongly palmitoylated during both vegetative hyphal growth and conidiation when constitutively expressed alone. This posttranslational lipid modification correlates the endosomal localization of all early melanin enzymes. Intriguingly, bioinformatic analyses predict that palmitoylation is a common mechanism for potential membrane association of polyketide synthases (PKSs) and nonribosomal peptide synthetases (NRPSs) in *A. fumigatus*. Our findings indicate that protein-protein interactions facilitate melanization by metabolic channeling, while posttranslational lipid modifications help recruit the atypical enzymes to the secretory pathway, which is critical for compartmentalization of secondary metabolism.

## IMPORTANCE

Subcellular compartmentalization is increasingly recognized as an important aspect of fungal secondary metabolism. It facilitates sequential enzymatic reactions, provides mobility for enzymes and metabolites, and offers protection against self-toxification. However, how compartmentalization is achieved remains unclear given that the majority of enzymes encoded by secondary metabolism gene clusters are predicted to be cytosolic proteins. Through studying melanization in *Aspergillus*, we previously found that all enzymes involved in the early steps of melanization are atypical secretory proteins. Here, we discovered physical interactions among melanin enzymes. However, it was the posttranslational palmitoylation rather than the physical interaction that was responsible for their recruitment to the secretory pathway. Intriguingly, palmitoylation is likely a common mechanism for potential membrane association of polyketide synthases (PKSs) and nonribosomal peptide synthetases (NRPSs) in *A. fumigatus*. Collectively, our findings suggest that posttranslational lipid modification helps direct secondary metabolism to defined organelles for biosynthesis and trafficking.

## INTRODUCTION

Melanins are pigmented biopolymers of indolic or phenolic precursors. Melanins contribute to species adaptation and survival by conferring resistance against UV and ionizing radiation, extreme temperatures, osmotic variations, reactive oxygen species (ROS), and biotic insults (1–4). In fungal species that are pathogenic to humans or plants, melanins are well-known virulence factors (5–7). For instance, the melanin coating on the conidia of the human pathogen *Aspergillus fumigatus* enhances fungal attachment to host tissues, helps *A. fumigatus* evade host immune recognition, scavenges host-generated ROS, inhibits macrophage apoptosis, and prevents phagolysosome fusion (8–11).

**FIG 1:**
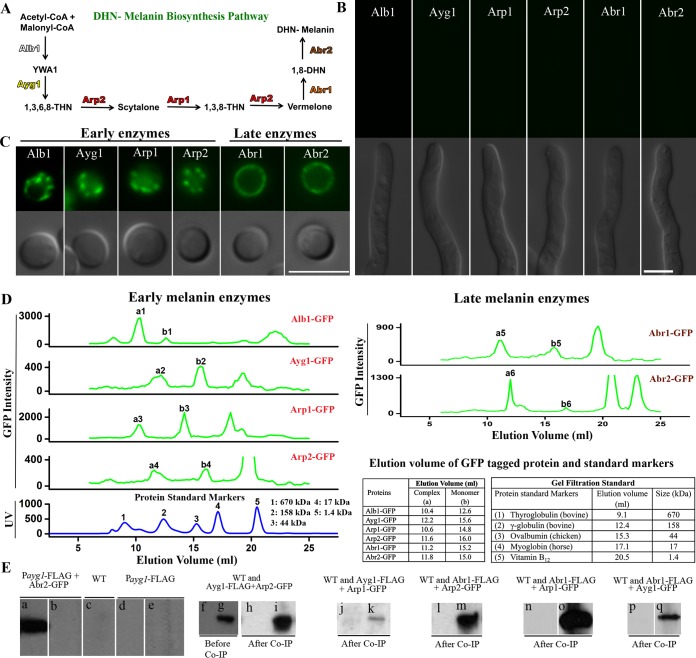
Interactions between the melanin enzymes. (A) A diagram of the melanin enzymatic pathway that involves four early enzymes and two late enzymes. CoA, coenzyme A. Six strains with Alb1-GFP, Ayg1-GFP, Arp2-GFP, Arp1-GFP, Abr1-GFP, and Abr2-GFP controlled by their native promoters were used in the experiments shown in panels B to D. (B) Melanin enzymes are not produced during vegetative hyphal growth based on the lack of GFP signal (top panel). Scale bar, 5 µm. (C) All 6 melanin enzymes are produced during conidiation based on the strong GFP signals (top panel). Early melanin enzymes are in endosomes, and late enzymes are mostly accumulated in the cell wall. Scale bar, 5 µm. (D) FPLC profiles of Alb1-GFP, Ayg1-GFP, Arp2-GFP, Arp1-GFP, Abr1-GFP, and Abr2-GFP, along with the standard marker protein profiles. The *y* axis indicates the relative florescence units (RFUs). (E) (a to e) Western blots were probed with GFP antibodies against the protein fraction pulled down by the anti-FLAG antibodies isolated from the P_*ayg1*_-FLAG+Arp2-GFP, untagged wild-type (WT), and P_*ayg1*_-FLAG (with only the FLAG tag) control strains. Images from before (a, c, and d) and after (b and e) Co-IP are shown. As expected, no signal was detected from any of the control strains after Co-IP (f, h, j, l, n, and p). The wild-type strain without any tag was used as a negative control (g and i). Signals were detected both before and after Co-IP using the strain with Ayg1-FLAG+Arp2-GFP. Similarly, signals were detected after Co-IP from strains expressing the following tagged proteins: Ayg1-FLAG+Arp1-GFP (k), Abr1-FLAG+Arp2-GFP (m), Abr1-FLAG+Arp1-GFP (o), and Abr1-FLAG+Ayg1-GFP (q).

The effectiveness of protection conferred by melanin depends on its biosynthesis, as well as its subcellular localization (12). In fungi, melanin is found in the cell wall as layers of globular particles, as well as in intracellular and extracellular vesicles (13–17). It is proposed that melanin is synthesized and trafficked through secretory vesicles. However, although some yeast species use classical secretory laccases to polymerize exogenously added precursors for melanization (14), the majority of filamentous fungi synthesize melanin *de novo* via the polyketide pathway (18). Like other secondary metabolism pathways, melanization through the polyketide pathway involves predicted cytosolic proteins, such as the polyketide synthase (PKS) and modification enzymes, as well as predicted conventional secretory laccases (19). For instance, the dihydroxynaphthalene (DHN) melanin biosynthesis pathway of *Aspergillus fumigatus* consists of six enzymes encoded by the melanin gene cluster (Fig. 1A). Based on the order of reactions that they carry out, the polyketide synthase Alb1 (also known as PksP) and the modification enzymes Ayg1, Arp1, and Arp2 are categorized as early enzymes, whereas the two laccases Abr1 and Abr2 are categorized as late enzymes (19). We demonstrated recently that the two late enzymes, laccases Abr1 and Abr2, are indeed secretory proteins and that they accumulate in the cell wall of conidiophores and conidia (19, 20). Surprisingly, contrary to the predicted cytoplasmic localization, all four early melanin enzymes are localized to the secretory endosomes (19). The discovery of all melanin enzymes trafficking to/through endosomes provides a plausible explanation for the subcellular compartmentalization of melanin biosynthesis and trafficking in secretory endosomes in *Aspergillus*.

The previous discovery indicates that the early melanin enzymes, including the PKS enzyme Alb1 and the modification enzymes Ayg1, Arp1, and Arp2, are “atypical” secretory proteins, given their lack of a signal peptide or a transmembrane domain. Here, we further surveyed the subcellular localization of six predicted cytoplasmic enzymes encoded by three different secondary metabolism gene clusters in *A. fumigatus*; namely, endocrocin, gliotoxin, and fumitremorigin B gene clusters. Our finding of these enzymes in vesicles, together with previously published studies on some enzymes in other gene clusters, suggests that atypical secretion is likely commonly used in secondary metabolism. Using melanization as a platform, this study was designed to investigate whether melanin enzymes from *A. fumigatus* formed complexes and whether their interaction was required for their atypical recruitment to the secretory pathway. Rather, our experiments demonstrated that the early melanin enzymes, including the foundation PKS enzyme Alb1, are palmitoylated and that such posttranslational lipid modification is critical for their endosomal localization. Intriguingly, bioinformatic analyses predict that all PKSs and nonribosomal peptide synthetases (NRPSs) in *A. fumigatus* are palmitoylated. Collectively, our findings indicate that atypical secretory proteins rely on posttranslational modifications to help compartmentalize secondary metabolism.

## RESULTS

### Interactions among melanin enzymes and their association with protein complexes.

The melanin gene cluster in *A. fumigatus* is known to be developmentally regulated at the transcriptional level, with induction of gene expression only during conidiation (19, 20). Consistent with the gene expression pattern, all of the functional green fluorescent protein (GFP)-tagged melanin enzymes expressed under the control of their native promoter were detected during conidiation (Fig. 1C) (19) but not during vegetative hyphal growth (Fig. 1B). The four early melanin enzymes (Alb1, Ayg1, Arp1, and Arp2) were localized to secretory vesicles in conidia (Fig. 1C) that were previously demonstrated to be endosomes (19). The two late laccase enzymes, Abr1 and Abr2, were secreted, and they delineated the conidia (Fig. 1C) due to their accumulation in the cell wall (19, 20).

Given that the early enzymes lack a signal peptide or a transmembrane domain, we hypothesize that they piggyback onto other secretory proteins in order to be recruited to the secretory pathway. If this is true, then the early enzymes will be associated with other secretory proteins and form complexes. We decided to examine this possibility through size exclusion chromatography. Here, we took advantage of six strains where each strain has one of the GFP-tagged melanin enzymes expressed under the control of its native promoter (Fig. 1B and C) (19). We extracted total proteins from young conidia and monitored the GFP signal of every fraction collected from fast protein liquid chromatography (FPLC). We detected a GFP signal peak from the fraction corresponding to the expected molecular mass of the single tagged protein in all six strains (Fig. 1D, Alb1-GFP, Ayg1-GFP, Arp1-GFP, Arp2-GFP, Abr1-GFP, and Abr2-GFP were detected as peaks b1 to b6, respectively). The expected size of each GFP-tagged melanin enzyme was verified by Western blotting. The unlabeled peaks corresponding to sizes smaller than the monomers were likely degraded GFP fusion proteins. Interestingly, we also detected a GFP signal from each fraction corresponding to a size much larger than that of the single protein (Fig. 1D, peaks a1 to a6). Thus, the results demonstrate that each of the six melanin enzymes is associated with a protein complex.

As all early enzymes are localized to endosomes (19), we hypothesize that early melanin enzymes might interact with each other. To test this hypothesis, we decided to perform coimmunoprecipitation assays (Co-IP) to examine whether one melanin enzyme interacts with another melanin enzyme. For this purpose, we constructed an *A. fumigatus* strain with FLAG-tagged Ayg1 and GFP-tagged Arp2. The expression of the tagged genes was under the control of their native promoters. We also generated two control strains, with one having the empty FLAG tag only and the other having Arp2-GFP and the empty FLAG tag. We extracted total proteins from these three strains and performed Co-IP experiments, using beads coated with anti-FLAG antibodies followed by Western blots probed with anti-GFP antibodies. As expected for the FLAG-only control, no Arp2-GFP signal was detected either before or after the pulldown (Fig. 1E, panels d and e). For the other control strain that carried the empty FLAG tag and Arp2-GFP, the Arp2-GFP signal was detected only before the pulldown and not after (Fig. 1E, panels a and b). The results suggest that there is no promiscuous interaction between the FLAG tag and GFP, or between Arp2 and the FLAG tag. For the strain with Ayg1-FLAG and Arp2-GFP, the Arp2-GFP signal was detected in both the total fraction and the FLAG tag pulldown fraction (Fig. 1E, panels g and i). The result indicates that these two early enzymes, Ayg1 and Arp2, interact with each other. Likewise, we detected interaction between Ayg1 and another early enzyme, Arp1 (Fig. 1E, panels j and k).

Given that early enzymes are recruited to endosomes and late enzymes traffic through endosomes before being secreted extracellularly (19), we hypothesize that late enzymes may interact with early enzymes, at least transiently. To test this hypothesis, we constructed an *A. fumigatus* strain with the late enzyme Abr1-FLAG and the early enzyme Arp2-GFP. We then performed Co-IP experiments using anti-FLAG antibodies as described above. We detected an Arp2-GFP signal from the Abr1-FLAG pulldown sample (Fig. 1E, panels l and m), indicating that the late enzyme Abr1 interacts with the early enzyme Arp2. Likewise, we detected interaction between the late enzyme Abr1 and two other early melanin enzymes tested, Ayg1 and Arp1 (Fig. 1E, panels n to q). Collectively, the findings indicate that there are interactions among early enzymes and also between early enzymes and late enzymes.

### The foundational PKS enzyme Alb1 is recruited to the secretory pathway during both conidiation and vegetative hyphal growth when expressed constitutively, while three other early enzymes, Ayg1, Arp1, and Arp2, are localized to vesicles only during conidiation.

**FIG 2:**
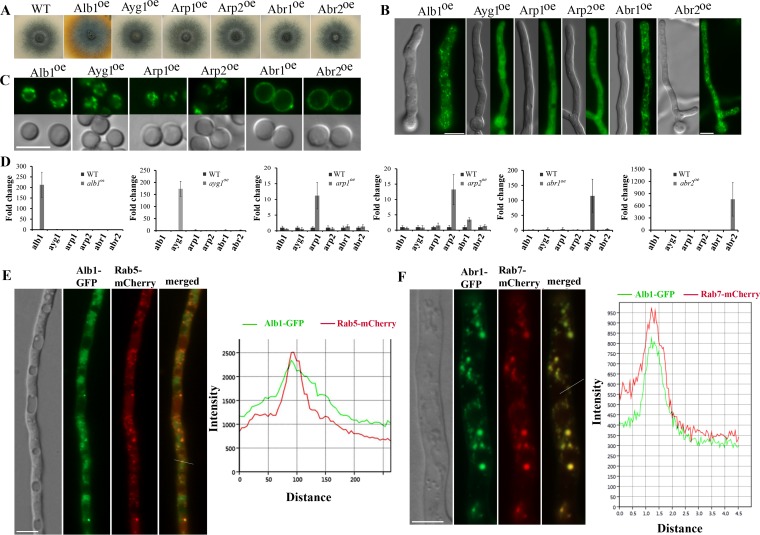
Distinct subcellular localization patterns among melanin enzymes when constitutively expressed alone. (A) Colony images of the overexpression strains P_*tef1*_-Alb1-GFP, P_*tef1*_-Ayg1-GFP, P_*tef1*_-Arp1-GFP, P_*tef1*_-Arp2-GFP, P_*tef1*_-Abr1-GFP, and P_*tef1*_-Abr2-GFP. All strains produced DHN melanin during conidiation, as evidenced by the bluish grey conidial color that is similar to that of the wild-type colony. (B) Fluorescence images of vegetative hyphae of all the melanin enzyme overexpression strains. (C) Fluorescence images of conidia of all the melanin enzyme overexpression strains. (D) Relative transcript levels of the six melanin genes *alb1, Ayg1, arp1*, *arp2, abr1*, and *abr2* in each of the melanin enzyme overexpression strains compared to that in WT during vegetative hyphal growth. Error bars show standard deviations. (E) Localization of the PKS enzyme Alb1-GFP and the endosomal marker Rab5-mCherry in vegetative hyphae (left), and a fluorescence intensity plot along a cellular axis indicated with a white line on the merged image (right). (F) Localization of the laccase Abr1-GFP and the endosomal marker Rab7-mCherry in vegetative hyphae (left), and a fluorescence intensity plot along a cellular axis indicated with a white line (right). The *y* axis used in panels E and F indicates the relative florescence units (RFUs).

As melanin enzymes are associated with protein complexes and early enzymes interact with late enzymes, it is tempting to speculate that the atypical secretory proteins (early enzymes) may piggyback onto the canonical secretory proteins (late enzymes) for their recruitment to the secretory pathway. If this piggyback hypothesis were valid, one would predict that each early melanin enzyme would become cytoplasmic when expressed alone. Because all melanin enzymes are coordinately upregulated during conidiation, it is challenging to examine the expression of any single melanin enzyme alone without the influence of other melanin enzymes. Hence, we took advantage of the fact that none of these enzymes were produced during vegetative hyphal growth (Fig. 1B), and we expressed each of the six GFP-tagged enzymes using the constitutively active *tef1*promoter. All six overexpression strains produced grayish blue melanin during conidiation, like the wild type (Fig. 2A), suggesting that overexpression of individual melanin enzymes did not impair melanization during conidiation. We noticed that overexpression of the PKS enzyme Alb1 led to the secretion of orange molecules into the medium during vegetative hyphal growth (Fig. 2A; see also Fig. S1 in the supplemental material). This was likely due to the ability of this polyketide synthase to supply precursors for multiple polyketides, as shown in *Aspergillus niger* (21). The constitutive expression of any one melanin gene did not show any significant impact on the transcript levels of other melanin genes during vegetative hyphal growth (Fig. 2D). During conidiation, all melanin genes were induced in these overexpression strains, as expected. Thus, these overexpression strains allowed us to examine the subcellular localization of individual melanin enzymes during vegetative hyphal growth without the interference of other melanin enzymes.

The two late enzymes, laccases Abr1 and Abr2, were still localized to the vesicles when they were constitutively expressed in vegetative hyphae (Fig. 2B). The vesicles highlighted by Abr1-GFP largely colocalized with the endosomal marker Rab7 (Fig. 2F). This result indicates that Abr1 and Abr2 can traffic to endosomes independently of other melanin enzymes, consistent with them being classified as canonical secretory proteins. It is interesting to note that Abr1 and Abr2 did not delineate the vegetative hypha cells as they did in conidia (Fig. 2B and C) (19, 20). The association of Abr1 and Abr2 with the cell wall in aerial cells was demonstrated previously through a plasmolysis assay, where the plasma membrane separated from the cell wall (19). In contrast, neither Abr1 nor Abr2 accumulated in the cell wall of vegetative hyphae (see Fig. S1 in the supplemental material). This difference between vegetative hyphae and conidiophore cell types could be due to the presence of hydrophobins (9) or other cell wall molecules uniquely present in aerial cells that trap extracellular Abr1 and Abr2 in the cell wall.

For the four early enzymes, two distinct localization patterns were observed. Three early enzymes, Ayg1, Arp1, and Arp2, showed diffused cytoplasmic localization when constitutively expressed alone in vegetative hyphae (Fig. 2B). However, they showed vesicular localization during conidiation (Fig. 2C). The observations suggest that the recruitment of the atypical enzymes Ayg1, Arp1, and Arp2 to the secretory pathway depends on other factors associated with conidiation. In sharp contrast to these three early enzymes, the constitutively expressed PKS Alb1 was localized to secretory vesicles in both vegetative hyphae and conidia (Fig. 2B and C). The vesicles decorated by Alb1 were also marked by the endosomal protein Rab5 (Fig. 2E). Therefore, this foundational enzyme can be recruited to the secretory pathway independently of other melanin enzymes.

**FIG 3:**
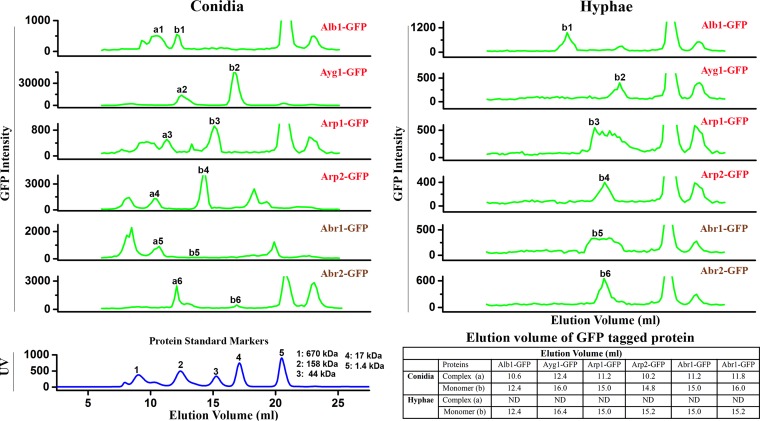
Constitutively expressed melanin enzymes are associated with protein complexes during conidiation but not during vegetative hyphal growth. FPLC profiles of the constitutively expressed melanin enzymes during vegetative hyphal growth (right) and during conidiation (left) are shown. The FPLC profiles are of P_*tef1*_-*alb1-GFP*, P_*tef1*_-*ayg1-GFP*, P_*tef1*_-*arp1-GFP*, P_*tef1*_-*arp2-GFP*, P_*tef1*_-*abr1-GFP*, and P_*tef1*_-*abr2-GFP* strains. The standard marker protein profiles (same as in Fig. 1D) are included here as a reference. The elution volumes used for graphing all the *x* axes are identical. The *y* axis indicates the relative florescence units (RFUs).

To examine whether the recruitment of these enzymes to the secretory pathway correlates with their association with protein complexes, we performed size exclusion chromatography for proteins extracted from vegetative hyphae and from young conidia of the six overexpression strains. None of the melanin enzymes formed detectable complexes when overexpressed alone in vegetative hyphae, based on the absence of GFP signals in fractions corresponding to sizes larger than those of each respective single protein alone (Fig. 3, right, peaks b1 to b6 represent the single proteins). In contrast, all six enzymes formed complexes in conidia, based on the presence of GFP signals in fractions corresponding to sizes much larger than those of each respective single protein alone (Fig. 3, left, peaks a1 to a6). The observation confirms our earlier results showing that the PKS enzyme Alb1 and the two conventional secretory proteins Abr1 and Abr2 could be sorted to the secretory pathway independently of other melanin enzymes.

### The recruitment of the early melanin enzymes, Ayg1, Arp1, and Arp2, to the secretory pathway does not require laccases Abr1/-2 or the PKS enzyme Alb1.

The observations described above indicate that the recruitment of the atypical enzymes Ayg1, Arp1, and Arp2 to the secretory pathway depends on other factors associated with conidiation. These factors could be other melanin enzymes naturally expressed during conidiation or other unknown factors. To our surprise, the deletion of both the *abr1* and the *abr2* gene in the P_*tef1*_-*ayg1-GFP* strain did not affect the vesicular localization of Ayg1 in conidia (see Fig. S2 in the supplemental material). Likewise, the deletion of both the *abr1* and the *abr2* gene did not affect the vesicular localization of Arp1 in conidia (see Fig. S2). Thus, the recruitment of these early melanin enzymes to the secretory pathway requires factors other than the two late melanin enzymes.

**FIG 4:**
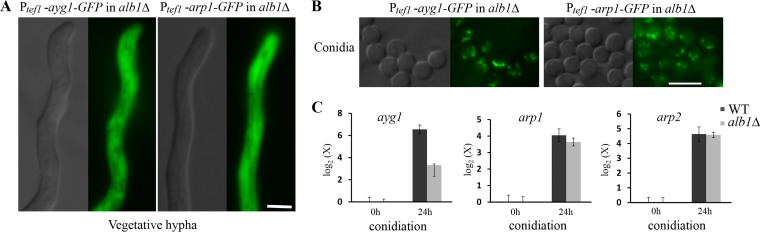
**T**he subcellular localization of the other early melanin enzymes, Ayg1 and Arp1, is independent of that of the PKS enzyme Alb1. (A and B) Localization of constitutively expressed Ayg1-GFP and Arp1-GFP in vegetative hyphae (A) and conidia (B) in the *alb1*Δ mutant background. Scale bar, 5 µm. (C) The relative transcript levels of *ayg1*, *arp1*, and *arp2* in the wild type and the *alb1*Δ mutant following induction during conidiation (24 h after transfer to solid medium). The transcript level of each gene was compared to that of the wild type during vegetative hyphal growth. Error bars show standard deviations.

The polyketide synthase Alb1 colocalizes with the remaining early enzymes in secretory endosomes (19), and this PKS can be recruited to the secretory pathway independently of other melanin enzymes. To test whether Alb1 helps recruit the other atypical early melanin enzymes, we decided to examine the subcellular localization of Ayg1 in the absence of the *alb1* gene. The deletion of the *alb1* gene did not impair the induction of expression of the *ayg1*, *arp1*, and *arp2* genes during conidiation (Fig. 4C). Overexpressed Ayg1 in the *alb1*Δ background, however, showed the same subcellular localization as Ayg1 in the wild-type background in both vegetative hyphae (Fig. 4A and 2B) and conidia (Fig. 4B and 2C). Similarly, the deletion of *alb1* did not affect the subcellular localization of Arp1-GFP (Fig. 4B). Thus, the trafficking of other early melanin enzymes does not depend on Alb1.

Taken together, the results indicate that the recruitment of other atypical melanin enzymes to the secretory pathway during conidiation does not require the polyketide synthase Alb1 or the two conventional secretory laccases, Abr1 and Abr2.

### The early melanin enzymes, including the polyketide synthase Alb1, are strongly palmitoylated, which is critical for proper subcellular localization.

One way that an atypical secretory enzyme could be recruited to the secretory pathway is through posttranslational lipid modifications. Using the GPS-Lipid (22) and the CSS-Palm (22, 23) prediction programs, we found potential palmitoylation sites in Arp2 and the foundational polyketide synthase, Alb1. Palmitoylation is a reversible lipid modification of proteins that plays important roles in protein trafficking to specific subcellular compartments (24–26).

**FIG 5:**
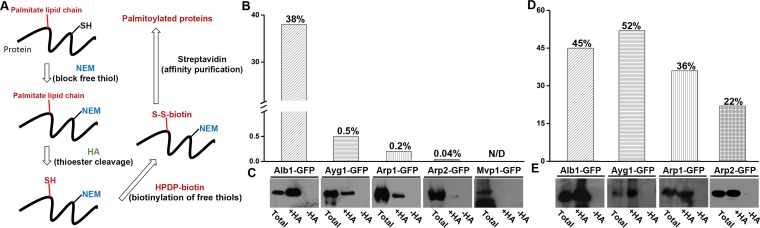
Detection of palmitoylated melanin enzymes in the overexpression strains. (A) A simplified flow chart for the experimental procedures to identify palmitoylated proteins. SH, thioester (free thiols); S-S-biotin, biotinylated disulfide. (B) Quantification of the ratios of recovered palmitoylated Alb1, Ayg1, Arp1, and Arp2 compared to the starting levels of the proteins was determined from total proteins extracted from vegetative hyphae of the P_*tef1*_-*alb1-GFP* strain, the P_*tef1*_-*ayg1-GFP* strain, the P_*tef1*_-*arp1-GFP* strain, and the P_*tef1*_-*arp2-GFP* strain. The P_*mvp1*_-*mvp1-GFP* strain was used as a control. (B) GFP-tagged melanin enzymes extracted from vegetative hyphae of strains used in the Western blot analysis whose results are shown in panel C were detected with anti-GFP antibody in the total protein fraction (starting total protein prior to the *in vitro* processing), the +HA group (the palmitoylated protein pool), and the −HA group (negative control for palmitoylation protein pool). Mvp1-GFP was not detected in the palmitoylated protein group. (D) GFP-tagged melanin enzymes extracted from conidia of strains used in the Western blot analysis whose results are shown in panel E were detected with anti-GFP antibody in the total protein fraction (starting total protein prior to *in vitro* processing), the +HA group (the palmitoylated protein pool), and the −HA group (negative control for palmitoylation protein pool). Mvp1-GFP was again not detected in the palmitoylated protein group.

As *in silico* prediction could be unreliable, we decided to identify palmitoylated conidial proteins in the wild-type strain by using chemical reporters that mimic the natural lipids to label those proteins. Because these chemical reporters also contain biotin as a bio-orthogonal chemical handle that reacts with streptavidin (27), the originally palmitoylated proteins can then be purified through streptavidin and identified by liquid chromatography-tandem mass spectrometry (LC-MS/MS) (Fig. 5A). By this approach, we identified 234 palmitoylated proteins present in conidia, with 99 palmitoylated proteins detected at levels of 6 or more peptides per protein (see Table S1 in the supplemental material). Among these 99 palmitoylated proteins are several G-protein subunits and Rho GTPases that are known to be palmitoylated in other organisms (28, 29), RasA that is an experimentally verified palmitoylated protein in *A. fumigatus* and *Cryptococcus neoformans* (30, 31), and chaperon proteins that are enriched in previous palmitoylation proteomics studies (32–34) (see Table S1).

Interestingly, three early melanin enzymes, namely, Alb1, Ayg1, and Arp2, were identified as palmitoylated conidial proteins. It is worth mentioning that Arp1, Arp2, and Ayg1, but not Alb1, are abundantly present in wild-type *A. fumigatus* conidia, based on an elegant quantitative proteomics study (35). Thus, the identification of Alb1 by palmitoylation profiling is possibly due to its strong palmitoylation. To examine the relative strength of palmitoylation of the melanin enzymes, we first extracted proteins using the same amounts of vegetative hyphal biomass from the P_*tef1*_-*alb1-GFP*, P_*tef1*_-*ayg1-GFP*, P_*tef1*_-*arp1-GFP*, P_*tef1*_-*arp2-GFP*, P_*tef1*_-*abr1-GFP*, and P_*tef1*_-*abr2-GFP* overexpression strains. Some of the total proteins extracted from each strain were then aliquoted equally, with one aliquot treated with the chemical hydroxylamine (+HA) for palmitoylated proteins and the other aliquot without the chemical treatment (−HA) (Fig. 5A). We then purified the proteins from both groups by using streptavidin beads as described above. Next, we separated the original total proteins, the purified palmitoylated proteins (+HA), and the purified proteins without the chemical treatment (−HA) on an SDS gel and probed for the melanin enzymes using the GFP antibody through Western blotting. As expected, no GFP signal was detected in any of the −HA groups (Fig. 5C). The two conventional secretory enzymes, Abr1 and Abr2, were not detected in the palmitoylated (+HA) group (see Fig. S2 in the supplemental material). No palmitoylation signal was detected for our negative control, Mvp1 (Fig. 5B). Mvp1 is an endosomal sorting nexin that localizes to endosomes by binding to phosphatidylinositol 3-phosphate enriched in endosomal membranes through its PHOX (PX) domain (19, 20, 36). The result is consistent with Abr1/-2 and Mvp1 not being identified by our palmitoylation profiling (see Table S1). In contrast, all the early melanin enzymes were recovered from the +HA groups that represented the palmitoylated pools (Fig. 5C). However, the ratios of recovered protein in the palmitoylated pool versus total protein for the four early enzymes were drastically different. Not surprisingly, the ratios of recovered palmitoylated protein compared to the original protein prior to the chemical treatment were 38% for Alb1, 0.5% for Ayg1, 0.2% for Arp1, and 0.04% for Arp2 (Fig. 5B). The calculated recovery rates are likely to be an underestimation due to unavoidable loss of yield in the experiment, which involved multiple chemical treatments and recovery steps. Nonetheless, these findings indicate that Alb1 is strongly palmitoylated, which offers a plausible explanation for its independent recruitment to the endosomes. The more-than-70-fold reduction in palmitoylation strength detected in other early melanin enzymes is likely insufficient to recruit these proteins to the endosomes, resulting in their cytoplasmic localization when expressed alone in vegetative hyphae.

We then examined the relative strength of palmitoylation of these constitutively expressed early melanin enzymes during conidiation using the same approach. All the early melanin enzymes were recovered from the +HA groups that represented the palmitoylated pools (Fig. 5E). The ratios of recovered palmitoylated protein compared to the original protein prior to the chemical treatment were 45% for Alb1, 52% for Ayg1, 36% for Arp1, and 22% for Arp2 (Fig. 5D). The proportions of the recovered palmitoylated proteins from the conidiating tissues, particularly for Ayg1, Arp1, and Arp2, were much higher than those from vegetative hyphae (Fig. 5D). The much higher levels of palmitoylation of the early melanin enzymes correlated with their endosomal localization in conidia. Collectively, the findings indicate that the constitutively expressed PKS enzyme Alb1 can be palmitoylated during both vegetative hyphal growth and conidiation. In contrast, the other early melanin enzymes (Ayg1, Arp1, and Arp2) are palmitoylated primarily during conidiation. The levels of their palmitoylation correlate with their subcellular localization.

**FIG 6:**
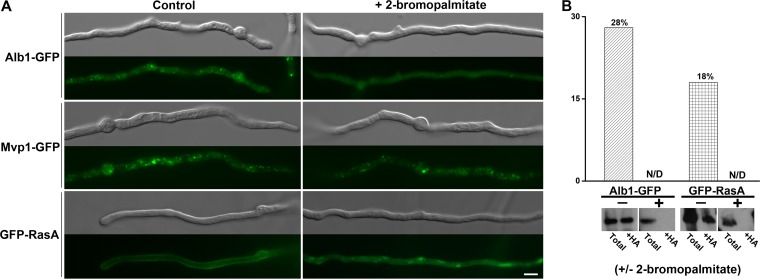
The impact of the palmitoylation inhibitor on Alb1-GFP. (A) Images of young hyphae of the P_*tef1*_-*alb1-GFP* strain, the P_*mvp1*_-*mvp1-GFP* strain, and the GFP-RasA-expressing strain with or without the palmitoylation inhibitor. Scale bar, 5 µm. (B) Quantification of the ratios of the recovered palmitoylated Alb1 and RasA compared to total proteins extracted from vegetative hyphae of the P_*tef1*_-*alb1-GFP* strain and the GFP-RasA-expressing strain before and after treatment with the palmitoylation inhibitor 2-bromopalmitate.

To further confirm the role of palmitoylation in Alb1’s endosomal localization, we tested the impact of the palmitoylation inhibitor 2-bromopalmitate (37, 38) on the subcellular distribution of Alb1. For a negative control, we used Mvp1, which localizes to endosomes through its PX domain. As expected, Mvp1 maintained its vesicular localization in the presence of this palmitoylation inhibitor (Fig. 6A). For a positive control, we used RasA, a known palmitoylated protein in *A. fumigatus* (30) that was also identified in our palmitoylation profiling (see Table S1 in the supplemental material). RasA localizes to the plasma membrane and endomembranes and can be lipid modified by both palmitoyl and farnesyl (30). Palmitoylation contributes to its plasma membrane localization (30). Indeed, we observed a reduced RasA signal from the plasma membrane in the presence of the palmitoylation inhibitor 2-bromopalmitate (Fig. 6A). When the P_*tef1*_-Alb1-GFP strain was treated with 2-bromopalmitate at 50 μM for 2 h, the Alb1 signal became weak and diffused in the cytoplasm (Fig. 6A). Consistent with this, treatment with 2-bromopalmitate reduced the palmitoylation of both Alb1 (28%) and RasA (18%) to nondetectable levels (Fig. 6B). Collectively, our findings indicate that palmitoylation is critical for the recruitment of the polyketide synthase Alb1 to the secretory pathway.

## DISCUSSION

The majority of secondary metabolism enzymes are predicted to be cytosolic, with no signal peptide, transmembrane domain, or cell wall anchor (see Table S2 in the supplemental material). For instance, none of the early melanin enzymes encoded by the melanin gene cluster are predicted to be secretory. Yet all early melanin enzymes are localized to endosomes in both *A. fumigatus* and *Aspergillus nidulans* (19). Similarly, none of the aflatoxin enzymes (Nor-1, Ver-1, and OmtA) are predicted to be secretory by SignalP or Wolf PSORT based on their sequences, and yet, they first appear in the cytoplasm and are then trafficked to endosomes/aflatoxisomes in *Aspergillus parasiticus* (39, 40). At least 13 aflatoxin enzymes, including most of the early aflatoxin enzymes, are present in the vesicle fraction based on a proteomic analysis (41), even though most of them are predicted to be cytosolic (see Table S2). To examine whether atypical trafficking is common for secondary metabolism, we examined the subcellular localization of six predicted cytoplasmic enzymes from the following three pathways by tagging them with GFP. These fluorescently labeled proteins were under the control of their native promoters. These enzymes include the PKS EncA and thioesterase EncB encoded by the endocrocin gene cluster, the aminotransferase GliI and cytochrome P450 GliC encoded by the gliotoxin gene cluster, and dimethyllacyl tryptophan synthase FumT and cytochrome P450 Fum-P450 encoded by the fumitremorigin B gene cluster. Remarkably, all of the enzymes tested showed vesicular localization (see Fig. S4). This finding further supports the emerging concept of subcellular compartmentalization of the biogenesis and trafficking of various polyketides and nonribosomal peptides (42–44). Therefore, atypical secretion is likely a common phenomenon in secondary metabolism.

We know little about how predicted cytoplasmic enzymes get recruited to the secretory pathway. Given that many secondary metabolism pathways, like melanization, require multiple enzymatic reactions and these genes are regulated in a coordinated fashion, it is not surprising that melanin enzymes physically interact with each other and form complexes. Forming a complex likely facilitates sequential enzymatic reactions, and it might also help stabilize proteins, as we previously showed that the Abr2 protein level was reduced when Abr1 was absent (19). Because many secondary metabolism pathways include both atypical secretory proteins and some conventional secretory proteins (see Table S2 in the supplemental material), it is plausible that forming a complex with conventional secretory proteins of the same pathway helps recruit the other proteins to the secretory pathway. Here, we found physical interactions among melanin enzymes and their association with protein complexes during conidiation when all other melanin enzymes are also produced. However, when these enzymes were expressed alone during vegetative hyphal growth, we could not detect their association with complexes. As expected for conventional secretory proteins, the two late enzymes were always localized to vesicles/endosomes. In contrast, the early melanin enzymes showed diffused cytoplasmic localization when expressed alone in vegetative hyphae, with the only exception being the polyketide synthase Alb1. Constitutively expressed Alb1 localized to endosomes during both vegetative hyphal growth and conidiation. However, contrary to our original hypothesis, the atypical secretion of Ayg1, Arp1, and Arp2 did not depend on the conventional secretory proteins Abr1 and Abr2 or palmitoylated Alb1.

By performing palmitoylation profiling of wild-type *A. fumigatus* proteins in conidia, we found that several melanin enzymes, including the PKS enzyme Alb1, are palmitoylated during conidiation (see Table S1 in the supplemental material). Examination of the constitutively expressed melanin enzymes from vegetative hyphae and from conidiating tissues revealed that all four early melanin enzymes (Alb1, Ayg1, Arp1, and Arp2) were strongly palmitoylated during conidiation, but only the PKS enzyme Alb1 was palmitoylated when expressed alone in vegetative hyphae. Their palmitoylation state offers a plausible explanation for their subcellular localization. Further investigation of the palmitoylation of Alb1 indicated that palmitoylation was critical for its endosomal localization. Our data so far indicate that the physical interactions between melanin enzymes may facilitate the sequential reactions required for melanin biosynthesis, but their recruitment to the secretory pathway and, consequently, the compartmentalization of melanization rely on the posttranslational lipid modification.

Intriguingly, all PKSs/NRPSs from *A. fumigatus* are predicted to be palmitoylated (see Fig. S5 in the supplemental material). As many secondary metabolism pathways are associated with the production of dormant survival structures like conidia or sclerotia (45), it is not unexpected that we only detected melanin enzymes and endocrocin enzymes by fluorescence microscopy in the wild type during conidiation (Fig. 1; see also Fig. S4 in the supplemental material) and not during vegetative hyphal growth. Based on the quantitative proteomic data from *A. fumigatus* (35), none of the 30 PKSs/NRPSs were detected from conidia, germination conidia, or young hyphae, with the exception of Alb1, which was detected in conidia with low confidence (35). This might explain why no other PKS enzymes beside Alb1 were detected in our palmitoylation profiling of conidial proteins (see Table S1). The low expression levels of PKSs/NRPSs make it challenging to investigate their subcellular localization and their posttranslation modifications. Nonetheless, as palmitoylation often delivers proteins to endosomes/lysosomes in diverse eukaryotes (24, 25), particularly in filamentous fungi (26), palmitoylation might constitute a common mechanism for recruiting atypical secondary metabolism enzymes to the secretory pathway.

## MATERIALS AND METHODS

### Strains, growth conditions, and terms.

The strains used in this study are listed in Table S3 in the supplemental material. Strains were grown on standard *Aspergillus* complete medium (CM) or minimal medium (MM) at 30°C with appropriate supplements as described previously (20). Terms used in this study are listed in Table S5.

### Protein tagging.

Strains with constitutive expression of Alb1-GFP, Ayg1-GFP, Arp1-GFP, Arp2-GFP, Abr1-GFP, and Abr2-GFP were generated using the *tef1* promoter to drive their expression. The GFP-tagged melanin gene constructs (*alb1*-GFP, *ayg1*-GFP, *arp1*-GFP, *arp2*-GFP, *abr1*-GFP, and *abr2*-GFP) were amplified from the previously reported strains expressing P_*alb1*_-Alb1-GFP, P_*ayg1*_-Ayg1-GFP, P_*arp1*_-Arp1-GFP, P_*arp2*_-Arp2-GFP, P_*abr1*_-Abr1-GFP, and P_*abr2*_-Abr2-GFP (19). The overexpression constructs (expressing P_*tef1*_-*alb1*-GFP, P_*tef1*_-*ayg1*-GFP, P_*tef1*_-*arp1*-GFP, P_*tef1*_-*arp2*-GFP, P_*tef1*_-*abr1*-GFP, and P_*tef1*_-*abr2*-GFP) were generated following the same approach as previously described (20). Likewise, strains in which P_*ayg1*_-Ayg1-GFP and P_*arp1*_-Arp1-GFP were constitutively expressed in the *alb1*Δ mutant background were generated. For Co-IP experiments, strains expressing Ayg1 and Abr1 with the FLAG tag fused at the C terminus were generated following the previously reported approach (19). 10×Gly::3×FLAG was amplified from a plasmid with GenBank accession number FJ457009 (46). The construct containing 10×Gly::3×FLAG flanked by the C terminus of the gene’s open reading frame (ORF) on one side and the downstream fragment of the gene’s ORF on the other was used to integrate the FLAG tag with the C terminus at the native gene locus. Integration of the FLAG tag at the native locus was verified through both PCR and Western blot analyses. To generate strains with Ayg1-FLAG and Arp2-GFP (Ayg1-FLAG+Arp2-GFP), Ayg1-FLAG+Arp1-GFP, Abr1-FLAG+Arp2-GFP, Abr1-FLAG+Arp2-GFP, Abr1-FLAG+Arp1-GFP, and Abr1-FLAG+Ayg1-GFP, the strains with FLAG-tagged Ayg1 or Abr1 were transformed with the P_*ayg1*_-*ayg1*-GFP, the P_*arp1*_-*arp1*-GFP, or the P_*arp2*_-*arp2*-GFP construct. Strains with a FLAG tag only (P_*tef1*_-FLAG) or a FLAG tag with GFP fused to Arp2 (P_*tef1*_-FLAG+Arp2-GFP) were generated and used as negative controls. The tagging of endosomal markers Rab7 and Rab5 with mCherry was performed as we described previously (19). The standard protoplasting approach was used for transformation as previously described (47).

### Construction of gene deletion mutants.

The procedure used to delete both the *abr1* gene and the *abr2* gene in the P_*tef1*_-*ayg1*-GFP strain and the P_*tef1*_-*arp1*-GFP strain background was essentially the same as we described previously (20). Briefly, a gene deletion construct that will replace both the *abr1* gene and the *abr2* gene (*abr1* and *abr2* are neighboring genes) was generated by fusion PCR as reported previously (48). The 2,232-bp hygromycin marker was fused with the 1.4-kb upstream fragment of the *abr2* open reading frame on one side and the 1.4-kb fragment containing the C-terminal region of the *abr1* gene, including the terminator, on the other side to generate the deletion construct.

### Gene expression analysis.

For gene expression studies, the wild type and the *alb1*Δ mutant were first grown vegetatively for 24 h in liquid medium. The vegetative hyphal mat was then transferred to the solid medium for synchronous conidiation as previously described (20). RNA samples were collected at the 0-h, 12-h, and 24-h time points posttransfer as we described previously (19). For gene expression studies during vegetative hyphal growth, RNA samples of the wild type and the melanin gene overexpression strains expressing P_*tef1*_-*alb1*-GFP, P_*tef1*_-*ayg1*-GFP, P_*tef1*_-*arp1*-GFP, P_*tef1*_-*arp2*-GFP, P_*tef1*_-*abr1*-GFP, and P_*tef1*_-*abr2*-GFP were collected after being cultured in liquid medium for 20 h. RNA samples were collected from three biologically independent experiments using the PureLink RNA minikit (Life Technologies) according to the manufacturer’s instructions. First-strand cDNA was synthesized with the SuperScript III cDNA synthesis kit (Invitrogen) according to the manufacturer’s instructions. Real-time PCR was performed using SYBR fast quantitative PCR (qPCR) master mix (KAPA Biosystems, Wilmington, MA). The house-keeping gene *tef1* was used to normalize the gene expression levels as we described previously (19).

### Microscopy and fluorescence.

Samples for microscopic observation were prepared the same way as previously described (49). Images were acquired and processed using a Zeiss Axioplan 2 imaging system with a Zeiss Imager M2 and an AxioCam 506 camera. GFP was visualized using filter set 38, HE GFP (Carl Zeiss Microscopy). The mCherry signal was visualized using filter set 43, HE Cy 3 (Carl Zeiss Microscopy).

### Protein extraction, Western blotting, and coimmunoprecipitation.

Proteins were extracted from conidia of strains with Ayg1-FLAG, Abr1-FLAG, Ayg1-GFP, Arp2-GFP, Arp1-GFP, Ayg1-FLAG+Arp2-GFP, Ayg1-FLAG+Arp1-GFP, Abr1-FLAG+Arp2-GFP, Abr1-FLAG+Arp2-GFP, Abr1-FLAG+Arp1-GFP, Abr1-FLAG+Ayg1-GFP, P_*tef1*_-FLAG+Arp2-GFP, and P_*tef1*_-FLAG. The tagged melanin proteins were all expressed using their native promoters. The protein extraction procedure was the same as previously described (50, 51). Aliquots of proteins were separated on SDS gels for Western blot analysis using the anti-FLAG (catalog number F2426; Sigma) or the anti-GFP (catalog number 11814460001; Roche) monoclonal antibody. Protein aliquots from FLAG-tagged strains were processed for Co-IP using anti-FLAG antibody following the manufacture’s protocol. The total cell lysate and Co-IP samples were then used to detect Arp2-GFP, Arp1-GFP, or Ayg1-GFP from the FLAG-tagged strains mentioned above. The Arp2-GFP signal was detected from total cell lysate of the FLAG-only+Arp2-GFP sample but not from its Co-IP sample. No Arp2-GFP signal was detected from either the total cell lysate or the Co-IP samples from the FLAG-only and the wild-type strain.

### Size exclusion chromatography.

Previously reported strains with GFP-tagged melanin genes controlled by their native promoters (P_*alb1*_-Alb1-GFP, P_*ayg1*_-Ayg1-GFP, P_*arp1*_-Arp1-GFP, P_*arp2*_-Arp2-GFP, P_*abr1*_-Abr1-GFP, and P_*abr2*_-Abr2-GFP) (19, 20) were used for size determination of all six melanin enzymes produced during conidiation. Conidia harvested from colonies cultured on MM solid medium for 2 days were used for protein extraction. Total proteins were extracted using the previously described method (50, 51)

For strains with constitutively expressed GFP-tagged melanin genes with the *tef1* promoter (P_*tef1*_-*alb1*-GFP, P_*tef1*_-*ayg1*-GFP, P_*tef1*_-*arp1*-GFP, P_*tef1*_-*arp2*-GFP, P_*tef1*_-*abr1*-GFP, and P_*tef1*_-*abr2*-GFP), proteins were extracted from both the vegetative hyphae and conidia. The vegetative hyphae were collected from overnight cultures in liquid MM. Conidia were harvested from colonies cultured on solid MM for 2 days. Vegetative hyphae or conidia were homogenized and resuspended in lysis buffer (25 mM HEPES, pH 7.5, 300 mM NaCl, 2 mM EDTA, 1% *n*-dodecyl-β-d-maltopyranoside [DDM], 1% NP-40, 1 mM phenylmethylsulfonyl fluoride [PMSF], 1 tablet proteinase cocktail inhibitor). Soluble proteins obtained by centrifugation were filtered first through a Spin-X filter column (Corning Costar) before being loaded onto a Superdex 200 10/300 GL column (GE Healthcare Life Sciences). The elution buffer (25 mM HEPES, pH 7.5, 300 mM NaCl, 2 mM EDTA, 1% DDM, 1% NP-40) was run through the column at a flow rate of 0.4 ml/min. The elution fraction was collected every 0.5 min (0.2 ml in volume) into an individual well, and each fraction was then measured for GFP fluorescence intensity with the Victor^3^V multilabel plate reader (PerkinElmer, Inc.). The FPLC profiles were generated by plotting the fluorescence intensity against the elution volume. Similarly, the gel filtration marker with standard proteins (catalog number 151-1901; BioRad) was loaded onto the same column with Superdex 200 10/300 GL. The profile was generated with UV intensity plotted against the elution volume.

### Palmitoylated protein extraction, protein identification with LC-MS/MS, and palmitoylation inhibitor.

The procedures for using the chemical reporter system were similar to those used to identify palmitoylated proteins in various organisms, including the fungus *Cryptococcus neoformans* (52, 53). Briefly, palmitoylated proteins were purified from conidia through acyl-biotin exchange following the same procedure as previously described (54, 55). The Cys-palmitoyl thioester linkages in the palmitoylated proteins were exchanged with biotin-HPDP {*N*-[6-(biotinamido)hexyl]-3'-(2'-pyridyldithio) propionamide}. The modified proteins were then purified using streptavidin cross-linked agarose beads. Briefly, the total proteins extracted from young conidia were processed by following a sequence of three *in vitro* chemical steps and one step of purification of the originally palmitoylated proteins with streptavidin, as follows. (i) The total proteins were extracted from conidia homogenized with a bead beater or mortar and pestle as described previously (50, 51). The proteins were resuspended in lysis buffer containing *N*-ethylmaleimide (NEM) (catalog number PI 23030; Pierce) overnight, which blocks the free thiols of the native proteins. The lysate was then precipitated with chloroform-methanol (CM), and the pellet was dissolved in 4SB buffer (4% SDS, 50 mM Tris-Cl, 5 mM EDTA, pH 7.4) and diluted 4 times with lysis buffer, following two more times of CM precipitation. (ii) The pellet from the last CM precipitation was dissolved in 4SB solution. The resuspension was divided into two aliquots. One aliquot was diluted with the lysis buffer, which contained 0.7 M hydroxylamine (HA; catalog number H330-100, Sigma) to cleave the bonds of Cys-palmitoyl thioester linkages present in the palmitoylated proteins (+HA group). The other aliquot was diluted with the same lysis buffer but without HA (−HA group) as the negative control. Both groups were then rotated for 1 to 3 h at 22°C and precipitated with three sequential CM treatments. (iii) The final pellet from the last CM precipitation was resuspended in 4SB buffer and then diluted with lysis buffer containing biotin-HPDP (catalog number PI 21341; Pierce). This step was to label free thiols that are newly released and exposed by the hydroxylamine treatment. The suspension with biotin-HPDP was rotated for 2 to 4 h at 22°C. Then, the suspension was precipitated and washed with three sequential CM precipitations to remove unbound biotin-HPDP. (iv) The pellet from the last CM precipitation was resuspended in 2SB buffer (2% SDS, 50 mM Tris-Cl, 5 mM EDTA, pH 7.4) and diluted 20 times with the lysis buffer. Insoluble particles were removed from the diluted suspension by centrifugation. Then, the suspension was incubated with the streptavidin cross-linked agarose beads (catalog number PI 20349; Pierce) at 22°C for 2 to 3 h with end-to-end rotation. Unbound proteins were removed by extensive washing with lysis buffer several times. Then, the purified proteins (originally palmitoylated) were eluted with 150 µl LB containing 0.1% SDS, 0.2% Triton X-100, and 1% β-mercaptoethanol (catalog number BP176; Fisher) (54). This approach allows the extraction of the originally palmitoylated proteins from the +HA group and not from the −HA group. Purified palmitoylated proteins were then identified with LC-MS/MS at both the Protein Chemistry Lab at Texas A&M University (http://tamupcl.com/) and the HSC Cores Research Facility at the University of Utah (http://cores.utah.edu/). The MS data were searched against the NCBI database.

To specifically examine the palmitoylation status of the melanin enzymes when they are expressed alone during vegetative hyphal growth and during conidiation, the hyphae or the conidiating tissues were collected from strains expressing the following individual constitutively expressed GFP-tagged melanin enzyme genes: P_*tef1*_-*alb1-GFP*, P_*tef1*_-*ayg1-GFP*, P_*tef1*_-*arp1-GFP*, P_*tef1*_-*arp2-GFP*, P_*tef1*_-*abr1-GFP*, and P_*tef1*_-*abr2-GFP*. These strains, together with the negative-control strain with P_*mvp1*_-*mvp1-GFP* and the positive-control strain expressing GFP-RasA, were cultured in liquid MM overnight. The total palmitoylated proteins from hyphae were extracted and purified as described above. Rather than LC-MS/MS, proteins from the −HA and +HA group were separated on the SDA gels and probed with GFP antibody to detect the presence and quantities of the GFP-labeled melanin enzymes. The original extracted proteins were run side by side on the gels with the +HA group and the −HA group. They were used to calculate the ratio of the recovered palmitoylated melanin enzymes present in the +HA group.

To test the impact of the inhibition of palmitoylation on the vesicular localization of the constitutively expressed Alb1-GFP, the P_*tef1*_-*alb1-GFP* strain was cultured in the liquid minimal medium for 16 h, washed, and then treated with fresh medium containing 2-bromopalmitate at 50 μM for 2 h (37). The control strains with P_*mvp1*_-*mvp1-GFP* (encoding the GFP-tagged endosomal-sorting nexin Mvp1) and the genes expressing GFP-RasA were processed under the same conditions.

## SUPPLEMENTAL MATERIAL

Figure S1  The two late melanin enzymes Abr1 and Abr2 are not accumulated in the cell wall during vegetative hyphal growth. (A) Images of the liquid cultures of the P_*tef1*_-Alb1-GFP and the P_*tef1*_-Abr1-GFP strains. Hyaline hyphae settled at the bottom of the flasks. (B) During plasmolysis of vegetative hyphae, the constitutively expressed Abr1-GFP remained intracellularly localized and was pulled away from the cell wall with the plasma membrane. (C) During plasmolysis of the conidiophore stalk, the constitutively expressed Abr1-GFP was associated with the cell wall and separated from the plasma membrane. Scale bar, 5 µm. Download Figure S1, TIF file, 3.1 MB

Figure S2  Two late melanin genes, *abr1* and *abr2*, are not required for the vesicular localization of the early melanin enzymes Ayg1 and Arp1. (A) Colony images of the wild type, the *abr1*Δ mutant, the *abr2*Δ mutant, and the P_*tef1*_-*Ayg1-GFP abr1*Δ *abr2*Δ mutant. (B) Fluorescence images of Ayg1-GFP and Arp1-GFP in vesicles during conidiation of the P_*tef1*_-*Ayg1-GFP abr1*Δ *abr2*Δ strain and the P_*tef1*_-*Arp1-GFP abr1*Δ *abr2*Δ strain. Download Figure S2, TIF file, 1.5 MB

Figure S3  Two late melanin enzymes, Abr1 and Abr2, are not palmitoylated. Constitutively expressed GFP-tagged melanin enzymes Abr1, Abr2, and Arp2 were probed with the anti-GFP antibody in the total protein fraction, the +HA group (the palmitoylated protein pool), and the −HA group (negative control) as described in the legend to Fig. 5. Download Figure S3, TIF file, 0.1 MB

Figure S4  Enzymes encoded by three different secondary metabolite gene clusters in *A. fumigatus* are localized to intracellular vesicles despite their predicted cytosolic localization. (A) Colocalization of EncA-GFP (PKS) and EncB-mCherry (thioesterase), encoded by the endocrocin gene cluster, in intracellular vesicles of *A. fumigatus* conidia. (B) Localization of GliI-GFP (aminotransferase) and GliC-GFP (cytochrome P450), encoded by the gliotoxin gene cluster, in intracellular vesicles. (C) Localization of FumT-GFP (dimethyllacyl tryptophan synthase) and Fum-P450-GFP (cytochrome P450), encoded by the fumitremorigin B gene cluster, in intracellular vesicles. The expression of all the fluorescently tagged proteins was driven by their native promoters. Download Figure S4, TIF file, 2.5 MB

Figure S5  The PKS or NRPS enzymes in *A. fumigatus* are predicted to be palmitoylated. The predicted palmitoylation score over the cutoff value is shown for each PKS (*n* = 13) or NRPS (*n* = 19, including PKS-NRPS hybrid) enzyme. For enzymes that have multiple potential palmitoylation sites, only the site with the highest score is shown in this graph. Yellow bar indicates the PKS enzyme for the endocrocin gene cluster. Red bar indicates the PKS enzyme for the melanin gene cluster. Green bar indicates the NRPS that is important for fumitremorgin biosynthesis. Blue bar indicates the NRPS in the gliotoxin gene cluster. Download Figure S5, TIF file, 0.2 MB

Text S1  Supplemental methods. Download Text S1, DOCX file, 0.02 MB

Table S1  Proteins identified by palmitoylation profiling from wild-type conidia. Table S1, DOCX file, 0.04 MB

Table S2  Prediction of subcellular localization of enzymes encoded by secondary metabolism gene clusters. Table S2, DOCX file, 0.01 MB

Table S3  Strains and plasmids used in this study. Table S3, DOCX file, 0.02 MB

Table S4  Primers used in this study. Table S4, DOCX file, 0.02 MB

Table S5  Terms used in this study. Table S5, DOCX file, 0.02 MB
