# Supplementary figures and images for: Interactions between Melanin Enzymes and Their Atypical Recruitment to the Secretory Pathway by Palmitoylation
Source: mBio. 2016 Nov 22;7(6):e01925-16. doi: 10.1128/mBio.01925-16 (PMC5120144; doi:10.1128/mBio.01925-16)

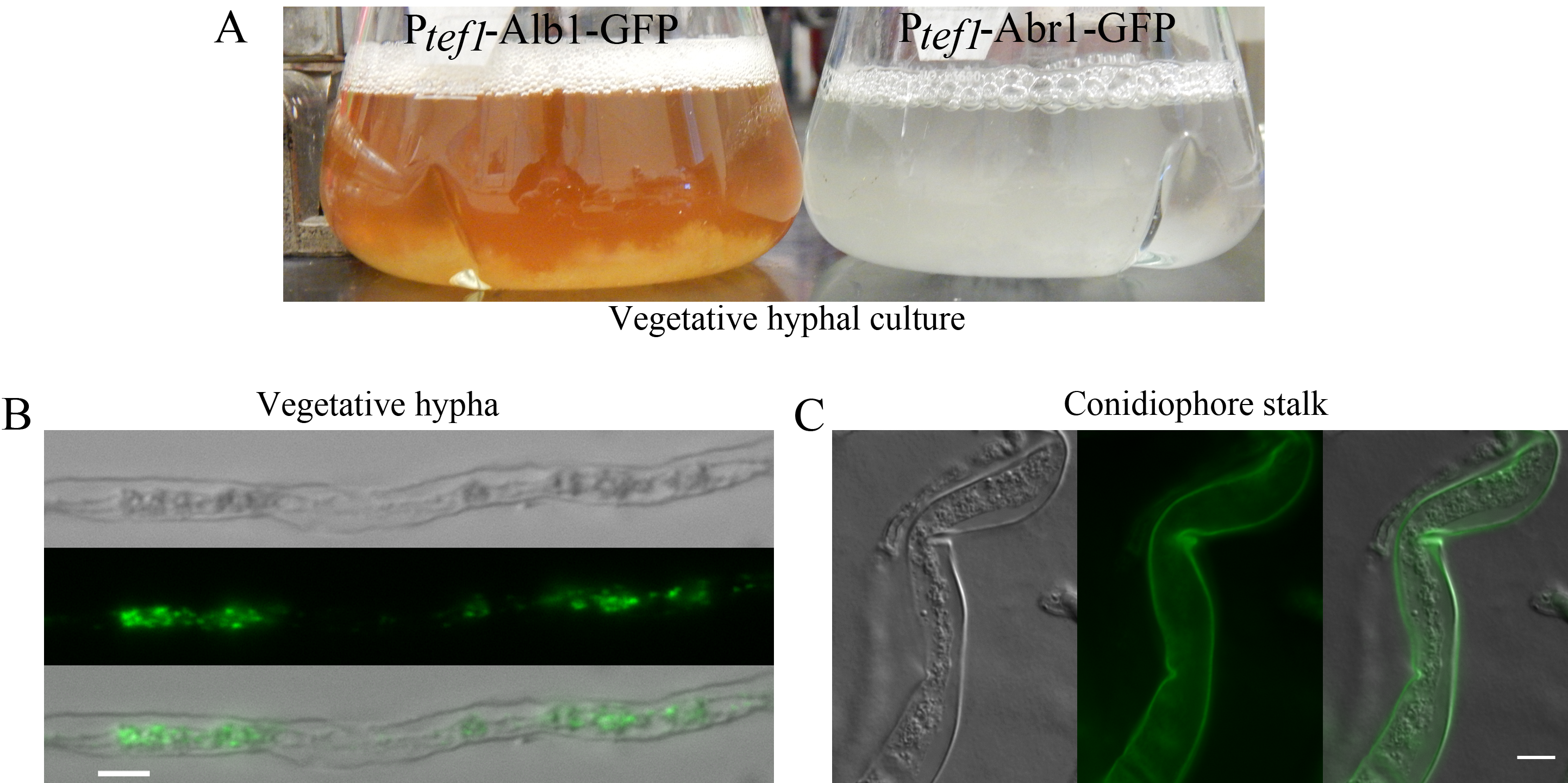

Supplement: Figure S1 — The two late melanin enzymes Abr1 and Abr2 are not accumulated in the cell wall during vegetative hyphal growth. (A) Images of the liquid cultures of the Ptef1-Alb1-GFP and the Ptef1-Abr1-GFP strains. Hyaline hyphae settled at the bottom of the flasks. (B) During plasmolysis of vegetative hyphae, the constitutively expressed Abr1-GFP remained intracellularly localized and was pulled away from the cell wall with the plasma membrane. (C) During plasmolysis of the conidiophore stalk, the constitutively expressed Abr1-GFP was associated with the cell wall and separated from the plasma membrane. Scale bar, 5 µm. Download [file mbo006163078sf1.tif]

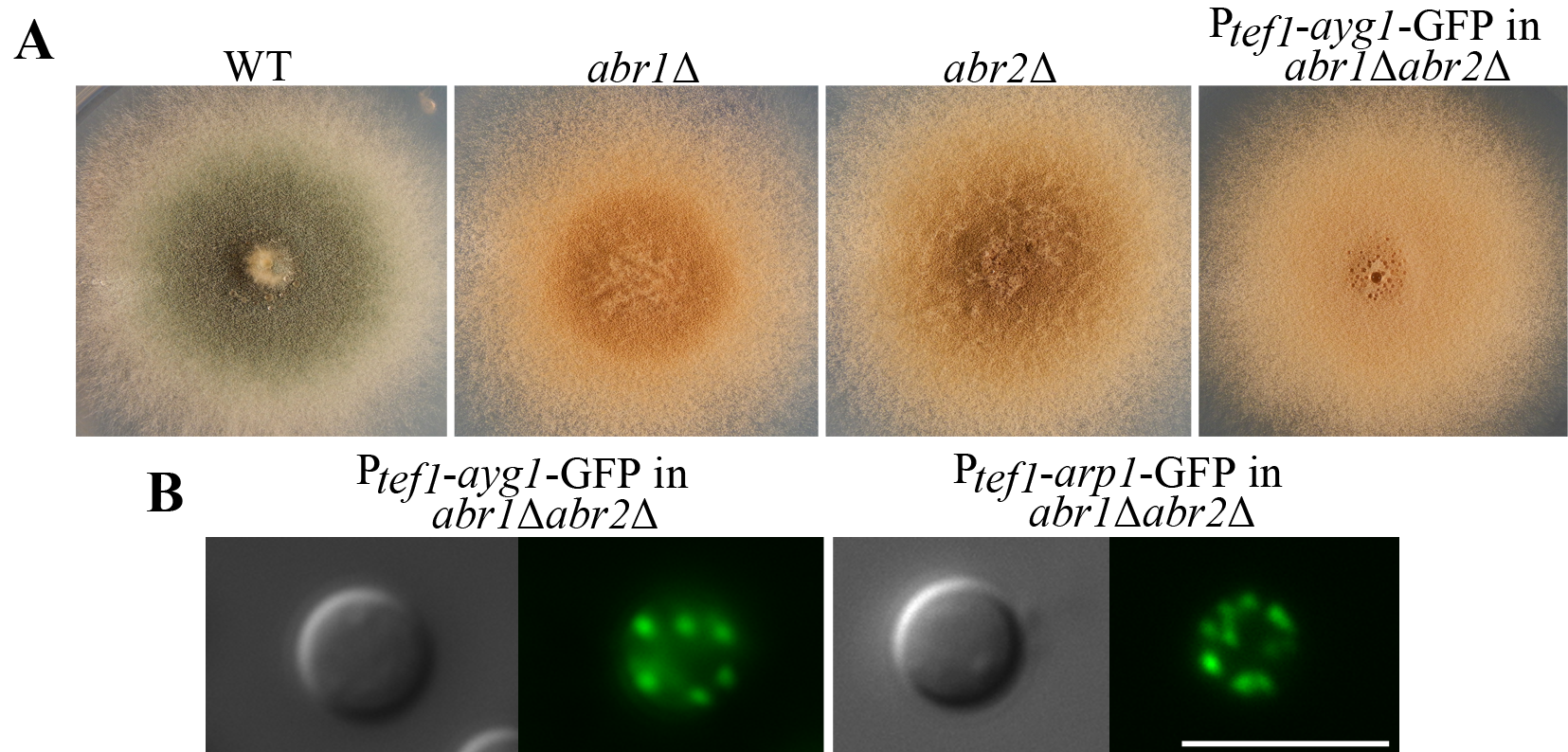

Supplement: Figure S2 — Two late melanin genes, abr1 and abr2, are not required for the vesicular localization of the early melanin enzymes Ayg1 and Arp1. (A) Colony images of the wild type, the abr1Δ mutant, the abr2Δ mutant, and the Ptef1-Ayg1-GFP abr1Δ abr2Δ mutant. (B) Fluorescence images of Ayg1-GFP and Arp1-GFP in vesicles during conidiation of the Ptef1-Ayg1-GFP abr1Δ abr2Δ strain and the Ptef1-Arp1-GFP abr1Δ abr2Δ strain. Download [file mbo006163078sf2.tif]

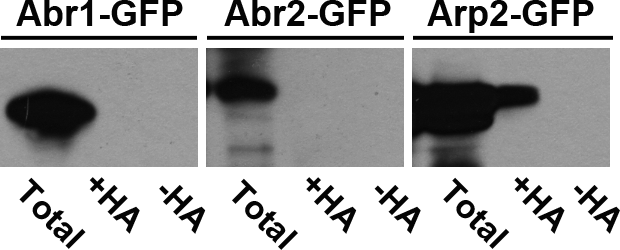

Supplement: Figure S3 — Two late melanin enzymes, Abr1 and Abr2, are not palmitoylated. Constitutively expressed GFP-tagged melanin enzymes Abr1, Abr2, and Arp2 were probed with the anti-GFP antibody in the total protein fraction, the +HA group (the palmitoylated protein pool), and the −HA group (negative control) as described in the legend to Fig. 5. Download [file mbo006163078sf3.tif]

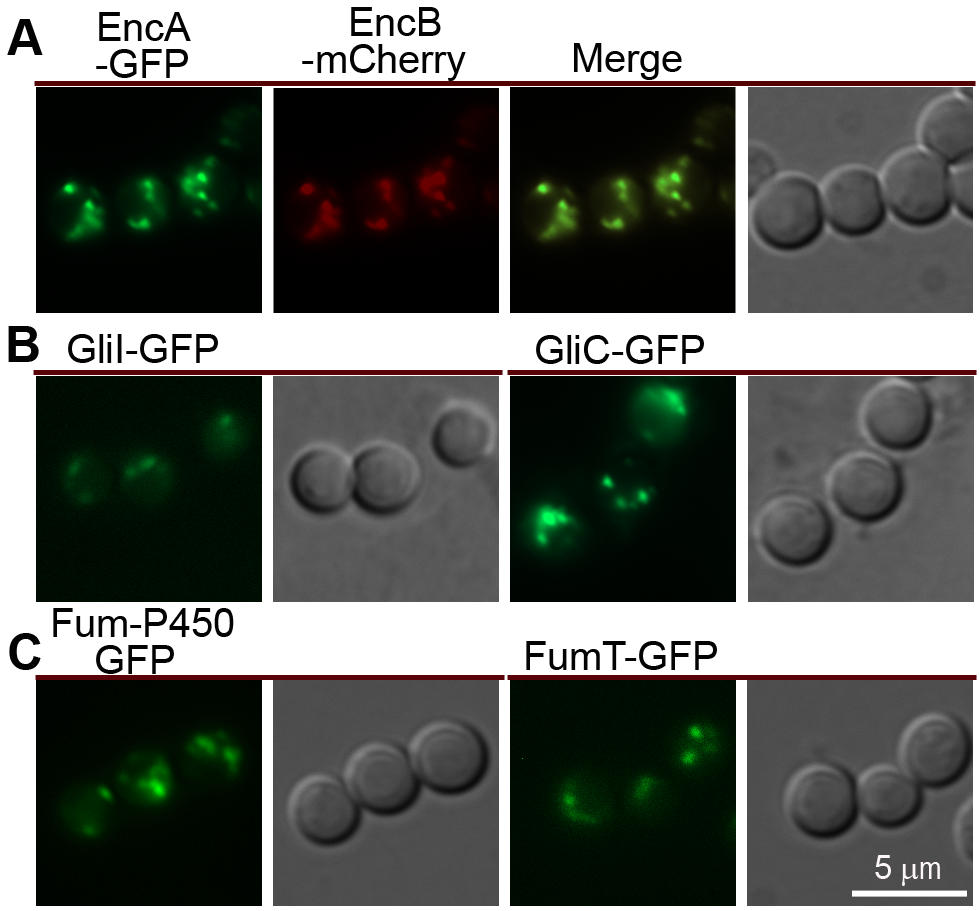

Supplement: Figure S4 — Enzymes encoded by three different secondary metabolite gene clusters in A. fumigatus are localized to intracellular vesicles despite their predicted cytosolic localization. (A) Colocalization of EncA-GFP (PKS) and EncB-mCherry (thioesterase), encoded by the endocrocin gene cluster, in intracellular vesicles of A. fumigatus conidia. (B) Localization of GliI-GFP (aminotransferase) and GliC-GFP (cytochrome P450), encoded by the gliotoxin gene cluster, in intracellular vesicles. (C) Localization of FumT-GFP (dimethyllacyl tryptophan synthase) and Fum-P450-GFP (cytochrome P450), encoded by the fumitremorigin B gene cluster, in intracellular vesicles. The expression of all the fluorescently tagged proteins was driven by their native promoters. Download [file mbo006163078sf4.tif]

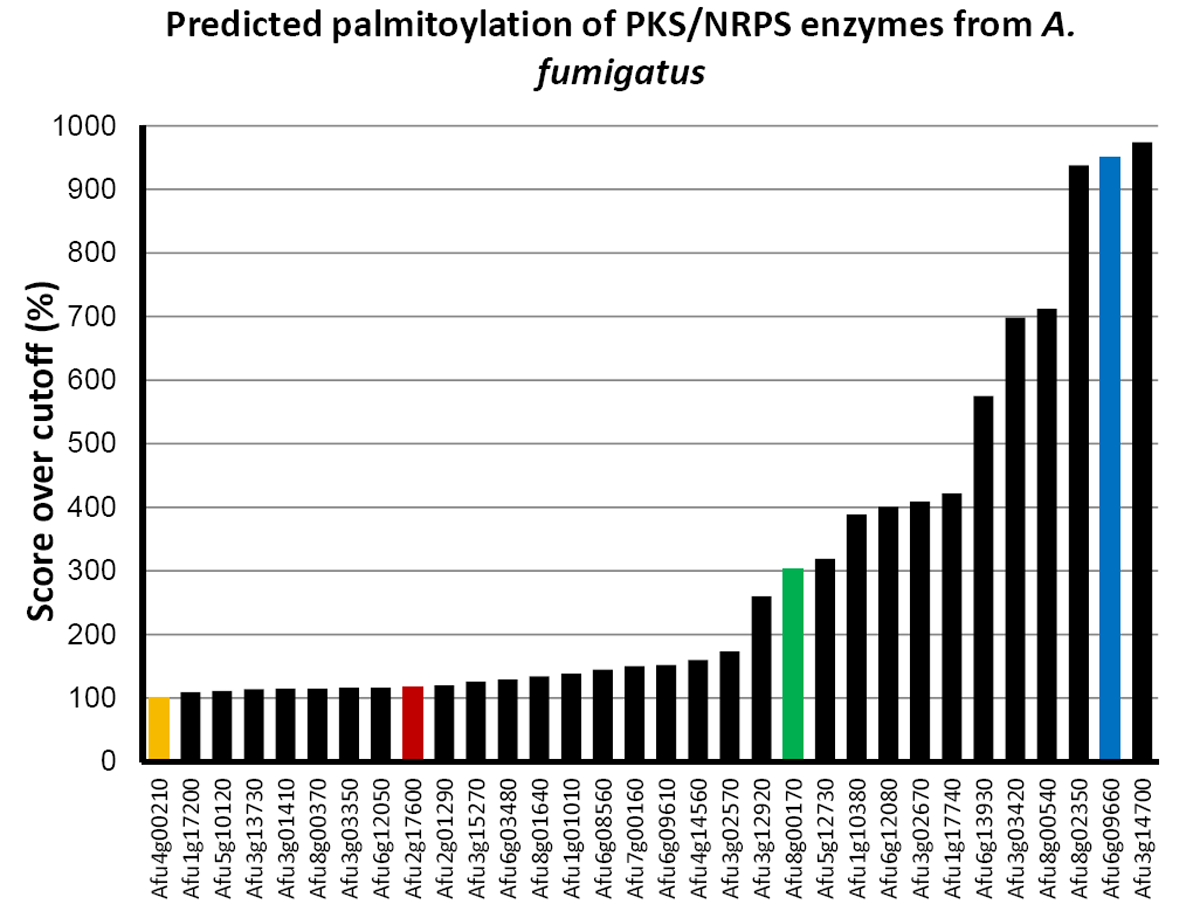

Supplement: Figure S5 — The PKS or NRPS enzymes in A. fumigatus are predicted to be palmitoylated. The predicted palmitoylation score over the cutoff value is shown for each PKS (n = 13) or NRPS (n = 19, including PKS-NRPS hybrid) enzyme. For enzymes that have multiple potential palmitoylation sites, only the site with the highest score is shown in this graph. Yellow bar indicates the PKS enzyme for the endocrocin gene cluster. Red bar indicates the PKS enzyme for the melanin gene cluster. Green bar indicates the NRPS that is important for fumitremorgin biosynthesis. Blue bar indicates the NRPS in the gliotoxin gene cluster. Download [file mbo006163078sf5.tif]
