# Supplementary material for: Interactions between Melanin Enzymes and Their Atypical Recruitment to the Secretory Pathway by Palmitoylation
Source: mBio. 2016 Nov 22;7(6):e01925-16. doi: 10.1128/mBio.01925-16 (PMC5120144; doi:10.1128/mBio.01925-16)
Supplement: Text S1 — Supplemental methods. Download [file mbo006163078s1.docx]

**Supplemental Methods**

**Fluorescence Tagging of Other Secondary Metabolism Enzymes.** For the prediction of secondary metabolism gene clusters, we used antiSMASH (antibiotics and Secondary Metabolite Analysis SHell) (1) and SMURF (Secondary Metabolite Unknown Regions Finder) algorithms (2). In total, 32 secondary metabolism gene clusters were identified by our predictions and the number of gene clusters identified is on par with previous predictions with multiple prediction softwares (3). We picked six enzymes from three different gene clusters and labelled with either GFP or mCherry. Two enzymes (EncA and EncB) from endocrocine gene cluster (4), two enzymes (GliI and GliC) from gliotoxin gene cluster (5), and two enzymes (FumT and FumP-450) from fumitremorgin (6). All the fluorescence tagged proteins were expressed using their native promoter following the same approach as we described previously (7, 8).

**Prediction of Palmitoylation of PKSs and NRPSs.** We used CSS-PALM (9) to predict palmitoylation sites of all PKS/NRPS generated from antiSMASH and SMURF. All PKS/NRPS possess at least one potential palmitoylation sites above the score cutoff. Although some of these proteins have potential sites for N-myristoylation, farnesylation, or geranylgeranylation, only palmitoylation sites are predicted in all of the 32 foundation enzymes (100%). In comparison, 35% of randomly selected 200 proteins encoded from chromosome 1 from Afu1g00010 to Afu1g02000 were predicted to be palmitoylated. Although this is possibly an overestimation, it does suggest that the PKS/NRPS enzymes are likely enriched for palmitoylation.

1. **Medema MH, Blin K, Cimermancic P, de Jager V, Zakrzewski P, Fischbach MA, Weber T, Takano E, Breitling R.** 2011. antiSMASH: rapid identification, annotation and analysis of secondary metabolite biosynthesis gene clusters in bacterial and fungal genome sequences. Nucleic Acids Res **39:**W339-346.

2. **Khaldi N, Seifuddin FT, Turner G, Haft D, Nierman WC, Wolfe KH, Fedorova ND.** 2010. SMURF: Genomic mapping of fungal secondary metabolite clusters. Fungal Genet Biol **47:**736-741.

3. **Inglis DO, Binkley J, Skrzypek MS, Arnaud MB, Cerqueira GC, Shah P, Wymore F, Wortman JR, Sherlock G.** 2013. Comprehensive annotation of secondary metabolite biosynthetic genes and gene clusters of Aspergillus nidulans, A. fumigatus, A. niger and A. oryzae. BMC Microbiology **13:**1-23.

4. **Lim FY, Hou Y, Chen Y, Oh JH, Lee I, Bugni TS, Keller NP.** 2012. Genome-based cluster deletion reveals an endocrocin biosynthetic pathway in Aspergillus fumigatus. Appl Environ Microbiol **78:**4117-4125.

5. **Scharf DH, Heinekamp T, Remme N, Hortschansky P, Brakhage AA, Hertweck C.** 2012. Biosynthesis and function of gliotoxin in *Aspergillus fumigatus*. Appl Microbiol Biotechnol **93:**467-472.

6. **Kato N, Suzuki H, Takagi H, Asami Y, Kakeya H, Uramoto M, Usui T, Takahashi S, Sugimoto Y, Osada H.** 2009. Identification of cytochrome P450s required for fumitremorgin biosynthesis in *Aspergillus fumigatus*. Chembiochem **10:**920-928.

7. **Upadhyay S, Torres G, Lin X.** 2013. Laccases involved in 1,8-dihydroxynaphthalene melanin biosynthesis in *Aspergillus fumigatus* are regulated by developmental factors and copper homeostasis. Eukaryot Cell **12:**1641-1652.

8. **Upadhyay S, Xu X, Lowry D, Jackson JC, Roberson RW, Lin X.** 2016. Subcellular compartmentalization and trafficking of the biosynthetic machinery for fungal melanin. Cell Rep **14:**2511-2518.

9. **Ren J, Wen L, Gao X, Jin C, Xue Y, Yao X.** 2008. CSS-Palm 2.0: an updated software for palmitoylation sites prediction. Protein Eng Des Sel **21:**639-644.
