# Supplementary material for: Interactions between Melanin Enzymes and Their Atypical Recruitment to the Secretory Pathway by Palmitoylation
Source: mBio. 2016 Nov 22;7(6):e01925-16. doi: 10.1128/mBio.01925-16 (PMC5120144; doi:10.1128/mBio.01925-16)
Supplement: Table S1 — Proteins identified by palmitoylation profiling from wild-type conidia. [file mbo006163078st1.docx]

| Accession Number | Identified Proteins | Molecular Weight | Peptides number detected |
| --- | --- | --- | --- |
| gi\|70986903 | pyruvate dehydrogenase complex, dihydrolipoamide acetyltransferase component | 52 kDa | 44 |
| gi\|70994626 | cobalamin-independent methionine synthase MetH/D | 87 kDa | 29 |
| gi\|159125523 | methylmalonate-semialdehyde dehydrogenase, putative | 64 kDa | 25 |
| gi\|70992355 | aldehyde dehydrogenase AldA | 61 kDa | 23 |
| gi\|159130150 | polyketide synthetase PksP | 234 kDa | 22 |
| gi\|71002010 | translation elongation factor EF-2 subunit | 93 kDa | 21 |
| gi\|146322501 | translation elongation factor EF-1 alpha subunit | 54 kDa | 19 |
| gi\|159125032 | pyruvate carboxylase, putative | 131 kDa | 19 |
| gi\|70989789 | protein disulfide isomerase Pdi1 | 56 kDa | 17 |
| gi\|70999113 | stomatin family protein | 37 kDa | 17 |
| gi\|70986914 | translation elongation factor eEF-3 | 118 kDa | 16 |
| gi\|159128078 | dihydrolipoamide succinyltransferase, putative | 48 kDa | 15 |
| gi\|159125085 | zinc-containing alcohol dehydrogenase, putative | 37 kDa | 15 |
| gi\|71002820 | conidial pigment biosynthesis 1,3,6,8-tetrahydroxynaphthalene reductase Arp2 | 29 kDa | 15 |
| gi\|42820760 | transketolase, putative | 75 kDa | 15 |
| gi\|70982606 | aldehyde dehydrogenase | 54 kDa | 15 |
| gi\|71002728 | cell division control protein Cdc48 | 90 kDa | 14 |
| gi\|70983360 | acetyl-CoA acetyltransferase | 41 kDa | 13 |
| gi\|70983614 | mitochondrial F1 ATPase subunit alpha | 60 kDa | 13 |
| gi\|70984800 | NADH-ubiquinone oxidoreductase | 73 kDa | 13 |
| gi\|70993866 | homocitrate synthase | 51 kDa | 13 |
| gi\|70992645 | mitochondrial aconitate hydratase | 86 kDa | 12 |
| gi\|70994244 | antioxidant protein LsfA | 25 kDa | 12 |
| gi\|70991695 | 6-phosphogluconate dehydrogenase Gnd1 | 56 kDa | 12 |
| gi\|70991282 | molecular chaperone Hsp70 | 70 kDa | 12 |
| gi\|159130145 | pigment biosynthesis protein Ayg1 | 45 kDa | 12 |
| gi\|70995858 | G-protein complex alpha subunit GpaA/FadA | 41 kDa | 12 |
| gi\|193783061 | glyceraldehyde-3-phosphate dehydrogenase | 36 kDa | 11 |
| gi\|70983346 | Hsp70 chaperone (HscA) | 67 kDa | 11 |
| gi\|70988990 | dihydrolipoamide dehydrogenase | 55 kDa | 11 |
| gi\|70999438 | phosphoglucomutase PgmA | 61 kDa | 11 |
| gi\|70997091 | vacuolar armadillo repeat protein Vac8 | 63 kDa | 10 |
| gi\|71000275 | succinate dehydrogenase subunit Sdh1 | 71 kDa | 9 |
| gi\|70984070 | malate dehydrogenase, NAD-dependent | 35 kDa | 9 |
| gi\|70982219 | succinyl-CoA synthetase beta subunit | 48 kDa | 9 |
| gi\|70995760 | 3-ketoacyl-CoA ketothiolase (Kat1) | 44 kDa | 9 |
| gi\|71000118 | inorganic diphosphatase | 44 kDa | 8 |
| gi\|70991443 | enolase/allergen Asp F 22 | 47 kDa | 8 |
| gi\|2072156 | actin | 42 kDa | 8 |
| gi\|70991589 | cell wall integrity signaling protein Lsp1/Pil1 | 39 kDa | 8 |
| gi\|159128092 | fatty acid synthase alpha subunit FasA, putative | 205 kDa | 8 |
| gi\|70990946 | vacuolar sorting receptor (Mrl1) | 38 kDa | 8 |
| gi\|70997968 | transaldolase | 35 kDa | 7 |
| gi\|70999940 | serine hydroxymethyltransferase | 52 kDa | 7 |
| gi\|70993772 | 3-ketoacyl-coA thiolase peroxisomal A precursor | 44 kDa | 7 |
| gi\|146324890 | Dyp-type peroxidase family protein | 56 kDa | 7 |
| gi\|70999137 | 40S ribosomal protein S0 | 32 kDa | 7 |
| gi\|146323885 | glutamine synthetase | 40 kDa | 7 |
| gi\|70999324 | G protein complex alpha subunit GanA | 41 kDa | 7 |
| gi\|70993620 | glycerol dehydrogenase (GldB), putative | 37 kDa | 7 |
| gi\|159128905 | IMP dehydrogenase, putative | 58 kDa | 7 |
| gi\|70991575 | pyruvate kinase | 58 kDa | 7 |
| gi\|70994774 | Glutamate/Leucine/Phenylalanine/Valine dehydrogenase | 49 kDa | 7 |
| gi\|159125120 | aldehyde dehydrogenase Ald3, putative | 60 kDa | 7 |
| gi\|70982442 | UTP-glucose-1-phosphate uridylyltransferase Ugp1, putative | 58 kDa | 7 |
| gi\|70990724 | alcohol dehydrogenase, zinc-containing | 38 kDa | 6 |
| gi\|70999466 | fructose-bisphosphate aldolase, class II | 40 kDa | 6 |
| gi\|70983971 | AhpC/TSA family thioredoxin peroxidase, putative | 30 kDa | 6 |
| gi\|146322787 | argininosuccinate synthase | 46 kDa | 6 |
| gi\|1843578 | catalase | 85 kDa | 6 |
| gi\|70994716 | outer mitochondrial membrane protein porin | 30 kDa | 6 |
| gi\|70991272 | glutamate synthase Glt1 | 234 kDa | 6 |
| gi\|70989229 | 14-3-3 family protein ArtA, putative | 29 kDa | 6 |
| gi\|70991439 | 14-3-3 family protein | 30 kDa | 6 |
| gi\|70996256 | mitochondrial phosphate carrier protein (Mir1) | 34 kDa | 6 |
| gi\|71002676 | curved DNA-binding protein (42 kDa protein) | 44 kDa | 6 |
| gi\|70997565 | RAS small monomeric GTPase RasA | 24 kDa | 6 |
| gi\|70995164 | GTP-binding protein YchF | 43 kDa | 6 |
| gi\|146323603 | Asp hemolysin-like protein | 16 kDa | 6 |
| gi\|70990878 | mitochondrial ADP,ATP carrier protein (Ant) | 33 kDa | 6 |
| gi\|70995790 | isocitrate dehydrogenase, NAD-dependent | 42 kDa | 6 |
| gi\|70984840 | molecular chaperone and allergen Mod-E/Hsp90/Hsp1 | 81 kDa | 6 |
| gi\|70991124 | predicted protein | 14 kDa | 6 |
| gi\|70995231 | adenosylhomocysteinase | 48 kDa | 5 |
| gi\|159122725 | NADP-dependent alcohol dehydrogenase | 36 kDa | 5 |
| gi\|71002394 | sorbitol/xylulose reductase Sou1-like | 28 kDa | 5 |
| gi\|159130510 | NADH-quinone oxidoreductase, putative | 22 kDa | 5 |
| gi\|71000162 | eukaryotic translation initiation factor 4, putative | 45 kDa | 5 |
| gi\|70997109 | triosephosphate isomerase | 28 kDa | 5 |
| gi\|70993334 | G-protein comlpex beta subunit CpcB | 35 kDa | 5 |
| gi\|225556722 | 40S ribosomal protein S3 [Ajellomyces capsulatus G186AR] | 30 kDa | 5 |
| gi\|146322408 | ATP synthase gamma chain, mitochondrial precursor | 32 kDa | 5 |
| gi\|70993752 | NADH-ubiquinone oxidoreductase, subunit F | 55 kDa | 5 |
| gi\|70984685 | allergen Asp F3 | 18 kDa | 5 |
| gi\|70997934 | carbonyl reductase | 31 kDa | 5 |
| gi\|70983023 | bifunctional catalase-peroxidase Cat2 | 84 kDa | 5 |
| gi\|70988765 | myo-inositol-phosphate synthase | 59 kDa | 5 |
| gi\|70990816 | 60S ribosomal protein P0 | 33 kDa | 5 |
| gi\|146323006 | dihydroxy acid dehydratase Ilv3 | 65 kDa | 5 |
| gi\|70997898 | conidial hydrophobin Hyp1/RodA | 16 kDa | 5 |
| gi\|5019414 | PPIase | 20 kDa | 5 |
| gi\|19577350 | probable 60S ribosomal protein l5 | 35 kDa | 5 |
| gi\|159131384 | Hsp70 chaperone Hsp88 | 80 kDa | 5 |
| gi\|70989741 | hexokinase Kxk, putative | 54 kDa | 5 |
| gi\|71002526 | karyopherin alpha subunit, putative | 61 kDa | 5 |
| gi\|159125171 | cytochrome c peroxidase Ccp1, putative | 40 kDa | 5 |
| gi\|159123033 | AIF-like mitochondrial oxidoreductase (Nfrl), putative | 60 kDa | 5 |
| gi\|70996142 | phosphoribosyl-AMP cyclohydrolase, putative | 93 kDa | 5 |
| gi\|70983492 | serine/threonine protein kinase | 47 kDa | 5 |
| gi\|71000509 | Lipoyltransferase | 33 kDa | 5 |
| gi\|71000136 | aspartyl aminopeptidase | 55 kDa | 5 |
| gi\|70992505 | isochorismatase family hydrolase | 21 kDa | 5 |
| gi\|70997740 | iron-sulfur protein subunit of succinate dehydrogenase Sdh2, putative | 34 kDa | 4 |
| gi\|70985386 | ThiJ/PfpI family protein | 25 kDa | 4 |
| gi\|70990636 | polyadenylate-binding protein | 81 kDa | 4 |
| gi\|41581286 | possible glycine cleavage system h protein | 13 kDa | 4 |
| gi\|159125358 | acetyl-coenzyme A synthetase FacA | 75 kDa | 4 |
| gi\|70986482 | pyruvate dehydrogenase E1 beta subunit PdbA, putative | 41 kDa | 4 |
| gi\|70993696 | saccharopine dehydrogenase Lys9 | 49 kDa | 4 |
| gi\|70992607 | dienelactone hydrolase family protein | 27 kDa | 4 |
| gi\|70989489 | ER Hsp70 chaperone BiP, putative | 73 kDa | 4 |
| gi\|70984695 | glucokinase GlkA, putative | 54 kDa | 4 |
| gi\|70995446 | 60S ribosomal protein L6 | 22 kDa | 4 |
| gi\|70995660 | translation elongation factor EF-Tu, putative | 48 kDa | 4 |
| gi\|70984647 | fumarate hydratase | 63 kDa | 4 |
| gi\|70994676 | SNARE domain protein | 31 kDa | 4 |
| gi\|159124214 | dynamin GTPase, putative | 93 kDa | 4 |
| gi\|70991463 | casein kinase I homolog, putative | 50 kDa | 4 |
| gi\|70982372 | UDP-N-acetylglucosamine pyrophosphorylase | 57 kDa | 4 |
| gi\|71001392 | 40S ribosomal protein S14 | 16 kDa | 4 |
| gi\|159126024 | SNARE domain protein | 28 kDa | 4 |
| gi\|70992209 | ATP-citrate synthase subunit 1 | 72 kDa | 4 |
| gi\|70997495 | Arp2/3 complex subunit (Arp3), putative | 48 kDa | 4 |
| gi\|70992505 | isochorismatase family hydrolase, putative | 24 kDa | 4 |
| gi\|70984134 | formate dehydrogenase | 46 kDa | 3 |
| gi\|71001436 | mannitol-1-phosphate dehydrogenase | 43 kDa | 3 |
| gi\|70996232 | 40S ribosomal protein S5 | 26 kDa | 3 |
| gi\|70995928 | spermidine synthase | 33 kDa | 3 |
| gi\|159122667 | dienelactone hydrolase family protein | 35 kDa | 3 |
| gi\|70982093 | 60S ribosomal protein L7 | 34 kDa | 3 |
| gi\|70990614 | Eukaryotic translation initiation factor eIF-5A | 21 kDa | 3 |
| gi\|70995281 | phosphoglycerate kinase PgkA | 45 kDa | 3 |
| gi\|71000467 | 40S ribosomal protein S4 | 29 kDa | 3 |
| gi\|70994557 | phosphoribosylaminoimidazolecarboxamide formyltransferase/IMP cyclohydrolase | 65 kDa | 3 |
| gi\|159125498 | conserved hypothetical protein | 102 kDa | 3 |
| gi\|70985224 | NAD dependent epimerase/dehydratase family protein | 35 kDa | 3 |
| gi\|70999822 | phosphatidylinositol transporter | 37 kDa | 3 |
| gi\|146324030 | alcohol dehydrogenase | 48 kDa | 3 |
| gi\|70997353 | disulfide isomerase (TigA) | 40 kDa | 3 |
| gi\|70990706 | NADH-cytochrome b5 reductase | 36 kDa | 3 |
| gi\|70984978 | nucleoside diphosphate kinase | 17 kDa | 3 |
| gi\|71000343 | peptidyl-prolyl cis-trans isomerase/cyclophilin | 18 kDa | 3 |
| gi\|70996112 | 60S ribosomal protein L17 | 23 kDa | 3 |
| gi\|70984206 | translation elongation factor eEF-1 subunit gamma | 54 kDa | 3 |
| gi\|70990790 | serine/threonine protein phosphatase PP1 | 37 kDa | 3 |
| gi\|159129289 | acetyl-CoA carboxylase | 255 kDa | 3 |
| gi\|159122928 | alcohol dehydrogenase, putative | 38 kDa | 3 |
| gi\|70982534 | thiosulfate sulfurtransferase | 38 kDa | 3 |
| gi\|159121936 | conserved hypothetical protein | 71 kDa | 3 |
| gi\|259487710 | TPA: ATP synthase beta chain, mitochondrial (Eurofung) [Aspergillus fumigatus Af293 FGSC A4] | 55 kDa | 3 |
| gi\|70993814 | AAA family ATPase Pontin, putative | 50 kDa | 3 |
| gi\|71001404 | 40S ribosomal protein S16 | 16 kDa | 3 |
| gi\|70993876 | aspartate aminotransferase | 48 kDa | 3 |
| gi\|224487982 | RecName: Full=Eukaryotic translation initiation factor 3 subunit E; Short=eIF3e | 52 kDa | 3 |
| gi\|70999540 | proteasome subunit alpha type | 28 kDa | 3 |
| gi\|70985092 | NAP family protein | 41 kDa | 3 |
| gi\|70998028 | 3-methylcrotonyl-CoA carboxylase subunit alpha (MccA) | 83 kDa | 3 |
| gi\|42820718 | smr family protein, putative | 28 kDa | 3 |
| gi\|70982516 | 40S ribosomal protein S2 | 28 kDa | 3 |
| gi\|169767532 | phosphoglycerate mutase, 2,3-bisphosphoglycerate-independent [Aspergillus oryzae RIB40] | 57 kDa | 3 |
| gi\|70989551 | conserved hypothetical protein | 48 kDa | 3 |
| gi\|53987049 | TPA_inf: glutamine:fructose-6-phosphate amidotransferase [Aspergillus fumigatus Af293 FGSC A4] | 77 kDa | 3 |
| gi\|70995400 | tubulin beta chain | 50 kDa | 3 |
| gi\|146323729 | eukaryotic translation initiation factor 2 gamma subunit | 56 kDa | 3 |
| gi\|70985384 | allergen, putative | 18 kDa | 3 |
| gi\|212542283 | mannose-1-phosphate guanylyltransferase [Talaromyces marneffei ATCC 18224] | 40 kDa | 3 |
| gi\|70985070 | 60S ribosomal protein L4 | 41 kDa | 3 |
| gi\|542191118 | NADP-glutamate dehydrogenase [Aspergillus terreus] | 49 kDa | 3 |
| gi\|70998202 | pyridoxine biosynthesis protein | 33 kDa | 3 |
| gi\|159124357 | 3'(2'),5'-bisphosphate nucleotidase | 44 kDa | 3 |
| gi\|70996414 | glutathione oxidoreductase Glr1 | 51 kDa | 3 |
| gi\|15375064 | plasma membrane H+-ATPase | 109 kDa | 2 |
| gi\|146322775 | aldehyde reductase | 33 kDa | 2 |
| gi\|70985198 | vacuolar ATP synthase catalytic subunit A | 75 kDa | 2 |
| gi\|70998911 | Ketol-acid reductoisomerase | 44 kDa | 2 |
| gi\|70984828 | citrate synthase (Cit1), putative | 52 kDa | 2 |
| gi\|70998544 | adenosine kinase | 37 kDa | 2 |
| gi\|71001164 | antigenic mitochondrial protein HSP60 | 62 kDa | 2 |
| gi\|70995022 | 60S ribosomal protein L9 | 22 kDa | 2 |
| gi\|159123405 | class III aminotransferase, putative | 51 kDa | 2 |
| gi\|71000507 | Rho GTPase Rho3 | 21 kDa | 2 |
| gi\|70989033 | actin cortical patch protein Sur7 | 27 kDa | 2 |
| gi\|159125861 | zinc-binding oxidoreductase, putative | 36 kDa | 2 |
| gi\|70994460 | succinate-semialdehyde dehydrogenase Uga2, putative | 58 kDa | 2 |
| gi\|70993054 | O-methyltransferase | 53 kDa | 2 |
| gi\|146323651 | copper resistance protein Crd2 | 11 kDa | 2 |
| gi\|146322509 | t-complex protein 1, eta subunit, putative | 61 kDa | 2 |
| gi\|70991873 | proteasome component Y13 | 28 kDa | 2 |
| gi\|159129149 | chromatin remodeling and histone acetyltransferase complexes subunit (Arp4) putative | 51 kDa | 2 |
| gi\|146322542 | UPF0160 domain protein MYG1 | 40 kDa | 2 |
| gi\|70991639 | alanine aminotransferase, putative | 54 kDa | 2 |
| gi\|159130844 | N-acetylglucosamine-phosphate mutase | 62 kDa | 2 |
| gi\|70999105 | translation initiation factor 2 alpha subunit, putative | 35 kDa | 2 |
| gi\|71001146 | 60S ribosomal protein L30 | 11 kDa | 2 |
| gi\|159131554 | Fasciclin domain family | 55 kDa | 2 |
| gi\|146322982 | NADH-ubiquinone oxidoreductase 49 kDa subunit, putative | 53 kDa | 2 |
| gi\|70985948 | GMC oxidoreductase, putative | 64 kDa | 2 |
| gi\|70996316 | adenylosuccinate synthetase AdB | 47 kDa | 2 |
| gi\|70995343 | S-adenosylmethionine synthetase | 42 kDa | 2 |
| gi\|70995816 | G protein complex alpha subunit GpaB | 41 kDa | 2 |
| gi\|70999736 | Rho GTPase Rho 2, putative | 22 kDa | 2 |
| gi\|31747203 | malate synthase | 60 kDa | 2 |
| gi\|70982362 | mRNA binding post-transcriptional regulator (Csx1), putative | 45 kDa | 2 |
| gi\|70990900 | 40S ribosomal protein S12 | 16 kDa | 2 |
| gi\|70990780 | proliferating cell nuclear antigen | 24 kDa | 2 |
| gi\|70993370 | thioredoxin reductase | 36 kDa | 2 |
| gi\|70998594 | succinyl-CoA synthetase alpha subunit, putative | 35 kDa | 2 |
| gi\|70986899 | malate dehydrogenase, NAD-dependent | 36 kDa | 2 |
| gi\|70993636 | alpha-ketoglutarate dehydrogenase complex subunit Kgd1, putative | 119 kDa | 2 |
| gi\|71000431 | 60S ribosomal protein L21 | 18 kDa | 2 |
| gi\|70985242 | proteasome component Pre6, putative | 30 kDa | 2 |
| gi\|70991361 | isocitrate dehydrogenase subunit 1, mitochondrial precursor | 42 kDa | 2 |
| gi\|70987245 | phospho-2-dehydro-3-deoxyheptonate aldolase | 40 kDa | 2 |
| gi\|70992245 | protein phosphatase 2a | 38 kDa | 2 |
| gi\|70992765 | 40S ribosomal protein S18 | 18 kDa | 2 |
| gi\|70997784 | NmrA-like family protein | 34 kDa | 2 |
| gi\|70992559 | AhpC/TSA family protein | 19 kDa | 2 |
| gi\|70998210 | aminopeptidase P | 73 kDa | 2 |
| gi\|70991162 | aconitate hydratase, mitochondrial | 85 kDa | 2 |
| gi\|70998961 | tRNA splicing protein (Spl1) | 57 kDa | 2 |
| gi\|159124326 | delta-1-pyrroline-5-carboxylate dehydrogenase PrnC | 63 kDa | 2 |
| gi\|70993228 | threonyl-tRNA synthetase | 84 kDa | 2 |
| gi\|146322361 | Phe-inhibited DAHP synthase AroG | 41 kDa | 2 |
| gi\|70994150 | aminopeptidase | 99 kDa | 2 |
| gi\|71002510 | prolyl-tRNA synthetase | 71 kDa | 2 |
| gi\|159124067 | O-methyltransferase, putative | 46 kDa | 2 |
| gi\|71000102 | 60S ribosomal protein L35Ae | 13 kDa | 2 |
| gi\|70990662 | 40S ribosomal protein S8 | 23 kDa | 2 |
| gi\|12230619 | Cu,Zn superoxide dismutase SOD1 | 16 kDa | 2 |
| gi\|70988705 | aldehyde dehydrogenase, putative | 52 kDa | 2 |
| gi\|70989809 | autophagy ubiquitin-activating enzyme ApgG | 80 kDa | 2 |
| gi\|70999520 | aspartic endopeptidase Pep2 | 44 kDa | 2 |
| gi\|70983656 | fumarate reductase Osm1 | 68 kDa | 2 |
| gi\|70989089 | aspartyl-tRNA synthetase Dps1, putative | 62 kDa | 2 |
