# Supplementary material for: Interactions between Melanin Enzymes and Their Atypical Recruitment to the Secretory Pathway by Palmitoylation
Source: mBio. 2016 Nov 22;7(6):e01925-16. doi: 10.1128/mBio.01925-16 (PMC5120144; doi:10.1128/mBio.01925-16)
Supplement: Table S2 — Prediction of subcellular localization of enzymes encoded by secondary metabolism gene clusters. [file mbo006163078st2.docx]

| **Supplemental Table 2. Prediction of subcellular localization of cluster proteins** | | | | | | | |
| --- | --- | --- | --- | --- | --- | --- | --- |
| Gene cluster | Total  Proteins | Regulator | Transporter | Enzymes | | | |
|  |  |  |  | Cytosol | Membrane | Organelle | Secreted |
| Penicillin G | 5 | 0 | 0 | 4 | 1 | 0 | 0 |
| Aflatoxin | 29 | 5 | 1 | 12 | 6 | 2 | 3 |
| Melanin* | 6 | 0 | 0 | 4 | 0 | 0 | 2 |
| Endocrocin* | 8 | 0 | 1 | 5 | 0 | 1 | 1 |
| Gliotoxin* | 13 | 1 | 1 | 8 | 0 | 1 | 2 |
| Fumitremorgin* | 15 | 2 | 0 | 5 | 4 | 3 | 1 |
| Sterigmatocystin | 25 | 1 | 0 | 10 | 4 | 6 | 4 |
| Fusaric Acid | 12 | 1 | 0 | 5 | 1 | 3 | 2 |
| *: cluster from *A. fumigatus*; Organelle: mitochondrion/peroxisome | | | | | | | |
