# Supplementary material for: Interactions between Melanin Enzymes and Their Atypical Recruitment to the Secretory Pathway by Palmitoylation
Source: mBio. 2016 Nov 22;7(6):e01925-16. doi: 10.1128/mBio.01925-16 (PMC5120144; doi:10.1128/mBio.01925-16)
Supplement: Table S3 — Strains and plasmids used in this study. [file mbo006163078st3.docx]

**Supplemental Table 2. Strains Used in This Study.**

| Strains | Genotype | Source |
| --- | --- | --- |
| CEA10 | Wild-type | FGSC |
| CEA17 | *pyrG1* | FGSC |
| ∆alb1.2 | *∆alb1::hph, AfupyrG2* | ([1](#_ENREF_1)) |
| AsuG4 | *pyrG1, P_alb1-_ alb1-eGFP-AfupyrG* | ([2](#_ENREF_2)) |
| AsuG6 | *pyrG1, P_ayg1-_ ayg1-eGFP-AfupyrG* | ([2](#_ENREF_2)) |
| AsuG8 | *pyrG1, P_arp1-_ arp1-eGFP-AfupyrG* | ([2](#_ENREF_2)) |
| AsuG9 | *pyrG1, P_arp2-_ arp2-eGFP-AfupyrG* | ([2](#_ENREF_2)) |
| AsuG1 | *pyrG1, P_abrA-_ abrA-eGFP-AfupyrG* | ([2](#_ENREF_2)) |
| AsuG3 | *pyrG1, P_abr2-_ abr2-eGFP-AfupyrG* | ([2](#_ENREF_2)) |
| AsuG10 | *pyrG1, P_mvp1-_ mvp1-eGFP-AfupyrG* | ([2](#_ENREF_2)) |
| Alb1OE | *pyrG1, P_tef1-_ alb1-eGFP-AfupyrG* | This study |
| Ayg1OE | *pyrG1, P _tef1-_ ayg1-eGFP-AfupyrG* | This study |
| Arp1OE | *pyrG1, P _tef1-_ arp1-eGFP-AfupyrG* | This study |
| Arp2OE | *pyrG1, P _tef1-_ arp2-eGFP-AfupyrG* | This study |
| Abr1OE | *pyrG1, P _tef1-_ abr1-eGFP-AfupyrG* | This study |
| Abr2OE | *pyrG1, P _tef1-_ abr2-eGFP-AfupyrG* | This study |
| AsuG20 | *∆alb1::hph, AfupyrG2, P _tef1-_ ayg1-eGFP-AfupyrG* | This study |
| AsuG21 | *∆alb1::hph, AfupyrG2, P _tef1-_ arp1-eGFP-AfupyrG* | This study |
| AsuF1 | *pyrG1, P_ayg1-_ ayg1-*3x*FLAG-hph* | This study |
| AsuF2 | *pyrG1, P_abr1-_ abr1-*3x*FLAG-hph* | This study |
| AsuF3 | *pyrG1, P_ayg1-_* 3x*FLAG-hph* | This study |
| AsuF4 | *pyrG1, P_ayg1-_* 3x*FLAG-hph, P_arp2-_ arp2-eGFP-AfupyrG* | This study |
| AsuF5 | *pyrG1, P_ayg1-_ ayg1-*3x*FLAG-hph, P_arp2-_ arp2-eGFP-AfupyrG* | This study |
| AsuF6 | *pyrG1, P_ayg1-_ ayg1-*3x*FLAG-hph, P_arp1-_ arp1-eGFP-AfupyrG* | This study |
| AsuF7 | *pyrG1, P_abr1-_ abr1-*3x*FLAG-hph, P_arp2-_ arp2-eGFP-AfupyrG* | This study |
| AsuF8 | *pyrG1, P_abr1-_ abr1-*3x*FLAG-hph, P_arp1-_ arp1-eGFP-AfupyrG* | This study |
| AsuF9 | *pyrG1, P_abr1-_ abr1-*3x*FLAG-hph, P_ayg1-_ ayg1-eGFP-AfupyrG* | This study |
| XX26 | *P_encA_-encA-GFP::pyrG,* | This study |
| XX46 | *P_fumP_-fumP450-GFP:: pyrG* | This study |
| XX47 | *P_fumT_-fumT-GFP::pyrG* | This study |
| XX106 | *P_gliI_-gliI-GFP::pyrG* | This study |
| XX108 | *P_gliC_-gliC-GFP::pyrG* | This study |
| XX257 | *P_encA_-encA-GFP::pyrG, PencB-encB-mCherry:: hph* | This study |
| AsuG22 | *P _tef1-_ ayg1-eGFP-AfupyrG, AfupyrG2, ∆abr1∆abr2::hph* | This study |
| AsuG23 | *P _tef1-_ arp1-eGFP-AfupyrG, AfupyrG2, ∆abr1∆abr2::hph* | This study |
| Alb1OER5 | *pyrG1, P_tef1-_ alb1-eGFP-AfupyrG, Prab5- mCherry-rab5, hph* | This study |
| Abr1OE47R7 | *pyrG1, P_tef1-_ alb1-eGFP-AfupyrG, P_rab7_-rab7:mCherry::hph* | This study |
| GFP-RasA |  | ([3](#_ENREF_3)) |

*Fungal Genetics Stock Center

1. **Upadhyay S, Torres G, Lin X.** 2013. Laccases involved in 1,8-dihydroxynaphthalene melanin biosynthesis in *Aspergillus fumigatus* are regulated by developmental factors and copper homeostasis. Eukaryot Cell **12:**1641-1652.

2. **Upadhyay S, Xu X, Lowry D, Jackson JC, Roberson RW, Lin X.** 2016. Subcellular compartmentalization and trafficking of the biosynthetic machinery for fungal melanin. Cell Rep **14:**2511-2518.

3. **Fortwendel JR, Juvvadi PR, Rogg LE, Asfaw YG, Burns KA, Randell SH, Steinbach WJ.** 2012. Plasma membrane localization is required for RasA-mediated polarized morphogenesis and virulence of *Aspergillus fumigatus*. Eukaryot Cell **11:**966-977.
