# Supplementary material for: Interactions between Melanin Enzymes and Their Atypical Recruitment to the Secretory Pathway by Palmitoylation
Source: mBio. 2016 Nov 22;7(6):e01925-16. doi: 10.1128/mBio.01925-16 (PMC5120144; doi:10.1128/mBio.01925-16)
Supplement: Table S4 — Primers used in this study. [file mbo006163078st4.docx]

Supplemental Table 3. Oligonucleotides Used in This Study.

| Primers | Sequence (5’🡪 3’) |
| --- | --- |
| TEF1Frtpcr | GTACCGGCAAGTCTGTTGA |
| TEF1Rrtpcr | ACGACACCAACAGCAACAG |
| alb1Frtpcr | TGAGCAGGATGATTCACAAC |
| alb1Rrtpcr | CAACTCCGAGTCGATAATCA |
| ayg1Frtpcr | CTCCATCGAGGACTTTGAG |
| ayg1Rrtpcr | GGGAGATGCGGTAGACAA |
| arp1Frtpcr | CGTCAAGACCCAGCATCT |
| arp1Rrtpcr | TCGACCTTGCGGTAGTAGT |
| arp2Frtpcr | ACTTCAACGAGGTGTTCAAT |
| arp2Rrtpcr | AATTGAGGACATGAGGATCA |
| abr1Frtpcr | TGAGCAGGATGATTCACAAC |
| abr1Rrtpcr | CAACTCCGAGTCGATAATCA |
| abr2Frtpcr | GTCTTCCAGCTGGTTGTCA |
| abr2Rrtpcr | TTGAGGTCCAGTTGAACTTG |
| FLAGF | GGCGGAGGCGGCGGAGGCGGA |
| FLAGR | GCGCGTTGGCCGATTCATTAAT |
| aygFLAGF1 | TTCGATACCGTCTTCCCGCACAA |
| aygFLAGR1 | TCCGCCTCCGCCGCCTCCGCCGTTCTTCGTCTTCGAAGGCG |
| aygFLAGF3 | ATTAATGAATCGGCCAACGCGCTCGAGACGTCGGTAGAGTCT |
| aygFLAGR3 | AAAGTCGACCGTCAGATCATCT |
| aygFLAGnesF | TAGAGGCCCTCTAATAGTCGTC |
| aygFLAGnesR | GTACACCATCAATGGGCAGA |
| abr1FLAGF1 | GACTCTGACCGACCATTACC |
| abr1FLAGR1 | CCGCCTCCGCCGCCTCCGCCCGAGGCATTTGCGCAGCCGT |
| abr1FLAGF2 | ACGGCTGCGCAAATGCCTCGGGCGGAGGCGGCGGAGGCGG |
| abr1FLAGR2 | GTCTATAAACAAGAAAGGAAGCGCGTTGGCCGATTCATTA |
| abr1FLAGF3 | TAATGAATCGGCCAACGCGCTTCCTTTCTTGTTTATAGAC |
| abr1FLAGR3 | TCCAAGCCGGCAAACACGAG |
| abr1FLAGnesF | ACATACCTCATCCACCTGGT |
| abr1FLAGnesR | TACGTGGACATTACCAAGCC |
| ayg1GFPF1 | CTGATCATTGCGCATCTGAA |
| aygFLAGR1-2 | CCGCCTCCGCCGCCTCCGCCCTTGATTACGTGCAGTGGTTT |
| aygFLAGF2-2 | AAACCACTGCACGTAATCAAGGGCGGAGGCGGCGGAGGCGG |
| ayg1GFPnesF | ACTGCGTGTACGGAGTGTAT |
| ayg1GFPnesR | AGTGAGCGCAACGCAATTAA |
| ayg1GFPnesF | ACTGCGTGTACGGAGTGTAT |
| arp1GFPnesF | GCCAAGAAGCCTTACTGAGT |
| arp2GFPnesF | TGATGGTGGCCAATGACCTC |
| GFPnesR | GGTCGACTTGTCCCTGAGGG |
| tefPF | GTAGCTGTTGATTATTTAGGCT |
| tefnesF | TGTAATTCTTACCGCCTGTC |
| albtefR1 | AGGCGATGGAGATCCTCCATTTTGACGGTTAGGTTTGAAC |
| albtefF2 | GTTCAAACCTAACCGTCAAAATGGAGGATCTCCATCGCCT |
| aygtefR1 | CCAAGGATCCAGCGTGGCATTTTGACGGTTAGGTTTGAAC |
| aygtefF2 | GTTCAAACCTAACCGTCAAAATGCCACGCTGGATCCTTGG |
| arp1tefR1 | TTGGGCTTCTTTTCGACCATTTTGACGGTTAGGTTTGAAC |
| arp1tefF2 | GTTCAAACCTAACCGTCAAAATGGTCGAAAAGAAGCCCAA |
| arp2tefR1 | TAGGTGCAGGTGTTCACCATTTTGACGGTTAGGTTTGAAC |
| arp2tefF2 | GTTCAAACCTAACCGTCAAAATGGTGAACACCTGCACCTA |
| abr1tefR1 | AGAGCCCTGGAATGGAACATTTTGACGGTTAGGTTTGAAC |
| abr2tefF2 | GTTCAAACCTAACCGTCAAAATGTTCCATTCCAGGGCTC |
| abr2tefR1 | AAATCAATAATATGACACATTTTGACGGTTAGGTTTGAAC |
| abr2tefF2 | GTTCAAACCTAACCGTCAAAATGTGTCATATTATTGATTT |
| GFPnesR2 | CTTCCATATTAAGCTATCAG |
| encAuF | AGCCGCTTTGTGGGCAGC |
| encAuR | CCAGCGCCTGCACCAGCTCC ACAATAATCAATCAACCAGGCTTTGAGATCAC |
| encAdF | CAGTGCCTCCTCTCAGACAG CACATGTTTTTCTTCTTCATTCCTCCGTGG |
| encAdR | CGATTCAGTCTAAACAGTTCTCCGTACGG |
| encAiF | TTCGAAAGCGAATTGCGCCTCCTG |
| encAiR | TCGCTCGCATCATCTCATTCCTGGAC |
| encBF | GCTTCAATCCAAGAAATTCCAGTGAGATC |
| encBR | CCAGCGCCTGCACCAGCTCCCCTACAAACCGGATTCTCTTGGCTC |
| gliIF | CCAGCGCCTGCACCAGCTCCGATAGCCGTCCATTTCTGCCCC |
| gliIR | GGTAGTGTTGCCAGGATTGACGG |
| gliCF | CCAGCGCCTGCACCAGCTCCATTCTTCTCGACCTCGCGTCCTAC |
| gliCR | AATAATCCAACCGAGCCACTGCGG |
| fumP450F | CCAGCGCCTGCACCAGCTCCCTCAAATCTACTCCGTCGCTGTCCATTC |
| fumP450R | GCGTATGCTCGCCGAAGTAGAAG |
| fumTF | CCAGCGCCTGCACCAGCTCCACCTCGAGTTGCTACATCCTCCC |
| fumTR | GTGGACCTGACGAATCCGGCATG |
| abr1abr2F1 | AGACTTGGGCGATGGTATGA |
| abr1abr2R1 | CACCACAATATATCCTGCCACCTGTTGTCGTGTATGAATG |
| abr1abr2F2 | TGGCAGGATATATTGTGGTG |
| abr1abr2R2 | ACCCGCCAATATATCCTGTC |
| abr1abr2F3 | GACAGGATATATTGGCGGGTAGACAAGAAGATCCGGCTG |
| abr1abr2F3 | TGCTGTGTCACTCTGGCTCA |
| abr1abr2nesF | TGATATGACTCTAGAGCCCG |
| abr1abr2nesR | ACTACGAGGCATTTGCGCAG |
