# Supplementary material for: Interactions between Melanin Enzymes and Their Atypical Recruitment to the Secretory Pathway by Palmitoylation
Source: mBio. 2016 Nov 22;7(6):e01925-16. doi: 10.1128/mBio.01925-16 (PMC5120144; doi:10.1128/mBio.01925-16)
Supplement: Table S5 — Terms used in this study. [file mbo006163078st5.docx]

**ST5. Terms used in this study**

| Terms | Definitions |
| --- | --- |
| Melanin | Biological pigmentation derived from polymerization of indolic or phenolic precursors |
| DHN | 1,8-dihydroxynaphthalene |
| Early enzymes | enzymes that function before the production of vermelone |
| Late enzymes | enzymes that function after the production of vermelone |
| Alb1 | conidial pigment polyketide synthase PksP, responsible for initiating synthesis of DNH-melanin, one of the early enzymes |
| PKS | Polyketide Synthase |
| Ayg1 | conidial pigment biosynthesis protein Ayg1, one of the early enzymes |
| Arp1 | conidial pigment biosynthesis scytalone dehydratase, one of the early enzymes |
| Arp2 | conidial pigment biosynthesis 1,3,6,8-tetrahydroxynaphthalene reductase, one of the early enzymes |
| Abr1 | conidial pigment biosynthesis oxidase, brown 1, one of the late enzymes |
| Abr2 | conidial pigment biosynthesis oxidase, Brown 2, one of the late enzymes |
| ROS | Reactive oxygen species |
| PX | Phox homology |
| Enc | Endocrocine |
| Gli | Gliotoxin |
| Fum | Fumitremorigin B |
| EncA | polyketide synthase for endocrocin, putative |
| EncB | metallo-beta-lactamase domain protein |
| GliI | aminotransferase |
| GliC | cytochrome P450 oxidoreductase |
| FumT | hexose transporter protein |
| FumP450 | monooxygenase P450 |
| 2-bromopalmitate | palmitoylation inhibitor |
| Mvp1 | Endosomal sorting nexin |
| Rab 5 | Small GTPase, used as an early endosomal marker |
| Rab 7 | small GTPase involved in endosomal maturation and vacuolar biogenesis, used as a late endosomal marker |
| Conventional secretion | classical secretion pathway, Proteins with signal peptide and traffickingthrough the route of ER and Golgi. |
| Unconventional secretion | non-typical secretion pathway. Proteins that do not have a signal sequence. They do not use the classical ER-Golgi pathway. |
| Atypical sorting | non-classic sorting |
| Co-IP | Co-Immunoprecipitation |
| MS | Mass Spectrometry |
| NRPS | nonribosomal peptide-synthetase |
| FPLC | Fast protein liquid chromatography |
| RasA | G-protein signaling GTPase, used as a positive control for palmitoylated proteins |
| HA | hydroxylamine, cleavage the palmitoylation chain |
| NEM | N-ethylmaleimide, blockage of free thiols |
